# On effects of elasticity and magnetic fields in the linear Rayleigh–Taylor instability of stratified fluids

**DOI:** 10.1186/s13660-018-1796-6

**Published:** 2018-08-02

**Authors:** Yuping Chen, Weiwei Wang, Youyi Zhao

**Affiliations:** 1 College of Mathematics and Computer Science, Fuzhou University, Fuzhou, China; 2 Key Laboratory of Operations Research and Control, Universities in Fujian, Fuzhou, China

**Keywords:** Stratified viscoelastic fluids, Rayleigh–Taylor instability, Stratified magnetohydrodynamic fluids

## Abstract

It is well known that there exists a threshold $\kappa_{{c}}$ such that the linearized stratified viscoelastic Rayleigh–Taylor problem is unstable for the elasticity coefficient *κ* satisfying $\kappa <\kappa_{{c}}$. In this paper, we further prove that if $\kappa <\kappa_{{c}}$, then there exists an unstable solution to the linearized stratified viscoelastic Rayleigh–Taylor problem with a largest growth rate. Moreover, the largest growth rate decreases from a positive constant to 0 as *κ* increases from 0 to $\kappa_{{c}}$. In addition, we further extend the obtained results in the linearized stratified viscoelastic Rayleigh–Taylor problem to the linearized stratified magnetic Rayleigh–Taylor problem.

## Introduction

Consider two completely plane-parallel layers of immiscible fluids, the heavier on top of the lighter one, and both subject to the Earth’s gravity. In this case, the equilibrium state is unstable to sustain small disturbances, and this unstable disturbance grows and leads to a release of potential energy, as the heavier fluid moves down under the gravitational force, and the lighter one is displaced upward. This unstable phenomenon was first studied by Rayleigh [21] and then by Taylor [22] and is called therefore the Rayleigh–Taylor (RT) instability. In the last decades, this phenomenon has been extensively investigated from both physical and numerical aspects; see [1, 4, 7, 9, 23] for examples. It has been also widely investigated how the RT instability evolves under the effects of other physical factors, such as elasticity [6, 17, 18], rotation [1, 3], internal surface tension [5, 25], magnetic fields [1, 2, 8, 10, 12, 15, 16], and so on.

Recently, Jiang et al. [17] established the nonlinear stability and linear instability of the stratified viscoelastic RT (VRT) problem, which models the motion of stratified immiscible viscoelastic fluids, the heavier on top of the lighter one, in the presence of a uniform gravitational field. Their results show that the elasticity can inhibit the development of RT instability. In this paper, we further investigate the effect of elasticity on the Rayleigh–Taylor instability based on the linear instability result of Jiang et al. Before stating our main result, we briefly introduce the stratified VRT problem and the results of Jiang et al.

The stratified VRT problem considered by Jiang et al. [17] reads as follows:

1.1 $$\left\{ \begin{array}{cc} \rho_{\pm }\partial_{t}v_{\pm }+\rho_{\pm }v_{\pm }\cdot \nabla v_{\pm }+\mathrm{div}S(p_{\pm }^{g},v_{\pm },U_{\pm })=0 & \text{in }\Omega_{\pm }(t),\\ \partial_{t}U_{\pm }+v_{\pm }\cdot \nabla U_{\pm }=\nabla v_{\pm }U_{\pm } & \text{in }\Omega_{\pm }(t),\\ \mathrm{div}v_{\pm }=0 & \text{in }\Omega_{\pm }(t),\\ d_{t}+v_{1}\partial_{1}d+v_{2}\partial_{2}d=v_{3} & \text{on }T,\\ 〚v_{\pm }〛=0,\;〚S(p_{\pm }^{g},v_{\pm },U_{\pm })〛\nu =gd〚\rho 〛\nu & \text{on }\Sigma (t),\\ v_{\pm }=0 & \text{on }\Sigma_{\pm },\\ v_{\pm }{|}_{t=0}=v_{\pm }^{0},U_{\pm }{|}_{t=0}=U_{\pm }^{0} & \text{in }\Omega_{\pm }(0),\\ d{|}_{t=0}=d_{0} & \text{on }T, \end{array}\right.$$

where $\mathcal{S}(p^{g}_{\pm},v_{\pm},U_{\pm}):=p^{g}_{\pm} I-\mu_{\pm}{S}(v_{\pm})-\kappa_{\pm}\rho_{\pm} (U_{\pm}U^{T}_{\pm}-I)$, $p^{g}_{\pm}:=p_{\pm} +g\rho_{\pm}x_{3}$, and $S(v_{\pm}):=\nabla v_{\pm}+\nabla v^{T}_{\pm}$. Next, we further explain the model and the notations appearing in (1.1) for reader’s convenience.

Equations (1.1)_1_–(1.1)_2_ describe the motion of the upper heavier and lower lighter viscoelastic fluids driven by the gravitational field along the negative $x_{3}$-direction, which occupy the two time-dependent disjoint open subsets $\Omega_{+}(t)$ and $\Omega_{-}(t)$ at time *t*, respectively. Equation (1.1)_3_ means that the fluids are incompressible. The two fluids interact with each other by the interfacial jump conditions (1.1)_5_ and the motion of a free interface (1.1)_4_, in which $d:=d(x_{1},x_{2},t)$ denotes the displacement function of the point at the interface deviating from the plane $\{x_{3}=0\}$. The nonslip boundary condition of the velocities on both upper and lower fixed flat boundaries are described by (1.1)_6_, and (1.1)_7_–(1.1)_8_ represent the initial status of the two fluids.

The notation $f_{+}$ and $f_{-}$ in (1.1) denote the values of the quantity *f* in the upper and lower fluids, respectively. In particular, the unknown functions $v_{\pm}:=v_{\pm}(x,t)\in\mathbb {R}^{3}$, $U_{\pm}(x,t)$, and $p_{\pm}(x,t)$ represent the velocity, the deformation tensor (a $3\times3$ matrix-valued function), and the hydrodynamic pressure of the two fluids, and $\rho_{\pm}$, $\mu_{\pm}$, and $\kappa_{\pm}$ denote the constant densities, viscosity coefficients, and elasticity coefficients of the two fluids, respectively. The positive constant *g*, the superscript *T*, and the capital letter *I* represent the gravitational constant, the transposition, and the $3\times3$ identity matrix. The notations $f^{0}$ or $f_{0}$ denote the initial data of *f*. In this paper, we consider that the domain Ω occupied by the two fluids is horizontal periodic, and thus we denote
1.2 where $\mathbb{T}_{i}:=2\pi L_{i}(\mathbb{R}/\mathbb{Z})$, $\mathbb {T}:=\mathbb{T}_{1}\times\mathbb{T}_{2}$, and $L_{1}$ and $L_{2}$ are positive constants. Moreover, we have the following expressions:

$$ \begin{aligned} &\Omega_{+}(t)=\bigl\{ (x_{h},x_{3})|x_{h} \in\mathbb{T}, d(x_{h},t)< x_{3}< m\bigr\} , \\ &\Omega_{-}(t)=\bigl\{ (x_{h},x_{3})|x_{h} \in\mathbb{T}, -l< x_{3}< d(x_{h},t)\bigr\} , \\ &\Omega=\Omega_{+}(t)\cup\Omega _{-}(t); \\ &\Sigma(t):=\bigl\{ \bigl(x_{h},d(x_{h},t) \bigr)|x_{h}\in\mathbb{T}\bigr\} , \qquad \Sigma_{-}:= \mathbb{T}\times\{x_{3}=-l\},\quad \mbox{and}\quad \Sigma_{+}:=\mathbb{T}\times\{x_{3}=m\}. \end{aligned} $$

Finally, we explain the interfacial jump conditions (1.1)_5_. The notation 〚⋅〛 stands for

$$〚f_{\pm }〛:=f_{+}{|}_{\Sigma (t)}-f_{-}{|}_{\Sigma (t)},$$

where $f_{\pm}|_{\Sigma(t)}$ are the traces of the quantities $f_{\pm }$ on $\Sigma(t)$. For two viscous fluids meeting at a free boundary, from the physical point of view, the velocity is continuous across the interface, and the jump in the normal stress is proportional to the mean curvature of the surface multiplied by the normal to the surface (see [14, 25]). Thus, we impose the jump conditions 〚v〛=0 on $\Sigma(t)$ and

$$〚S(p_{\pm },v_{\pm },U_{\pm })〛\nu =\vartheta C\nu \;\text{on }\Sigma (t),$$

where

1.3 $$ \begin{aligned} \mathcal{S}(p_{\pm},v_{\pm},U_{\pm}):=p_{\pm}I- \mu_{\pm} {S}(v_{\pm})-\kappa_{\pm}\rho_{\pm} \bigl(U_{\pm}U^{T}_{\pm}-I\bigr), \end{aligned} $$

and *ν* is the unit normal vector on $\Sigma(t)$, *ϑ* is the surface tension coefficient, and $\mathcal{C}$ is the twice the mean curvature of the internal surface $\Sigma(t)$. Since in this paper we focus on the elasticity effect upon the RT instability, we omit the surface tension and obtain therefore the second jump condition in (1.1)_5_. In addition, since the density of the upper fluid is heavier than the lower one, we have

$$〚\rho_{\pm }〛>0.$$

Problem (1.1) enjoys a stratified equilibrium state solution (i.e., stratified VRT equilibrium state): $(v,U,d,p^{g})=(0,I,\bar{d},\bar{p}^{g})$, where $\bar{d}\in(-l,m)$. We should point out that $\bar{p}^{g}$ can be uniquely computed out by hydrostatics, which depends on the variable $x_{3}$ and $\rho_{\pm}$ and is continuous with respect to $x_{3}\in (-l,m)$. Without loss of generality, we assume that $d = 0$. If *d̄* is not zero, we can adjust the $x_{3}$ coordinate to make $\bar{d}=0$. Thus *d* is regarded as the displacement away from the plane

$$ \Sigma:=\mathbb{T}\times\{0\}. $$

To simplify the representation of problem (1.1), we introduce the indicator function *χ* and denote

$$ \begin{aligned} &\rho=\rho_{+}\chi_{\Omega_{+}}+ \rho_{-}\chi_{\Omega_{-}}, \qquad \mu=\mu_{+} \chi_{\Omega_{+}}+\mu_{-}\chi_{\Omega_{-}}, \\ &\kappa=\kappa_{+}\chi_{\Omega_{+}}+\kappa_{-} \chi_{\Omega_{-}},\qquad v=v_{+}\chi_{\Omega_{+}}+v_{-} \chi_{\Omega_{-}}, \\ &U=U_{+}\chi _{\Omega_{+}}+U_{-}\chi_{\Omega_{-}}, \qquad p=p_{+}\chi_{\Omega _{+}}+p_{-}\chi_{\Omega_{-}}, \\ &v_{0}=v^{0}_{+}\chi_{\Omega_{+}}+v^{0}_{-} \chi_{\Omega_{-}}, \qquad U_{0}=U^{0}_{+} \chi_{\Omega_{+}}+U^{0}_{-}\chi_{\Omega_{-}}. \end{aligned} $$

Now, we denote the perturbation quantity to the equilibrium state $(0,I,0,\bar{p}^{g})$ by

$$ v=v-0,\qquad \sigma=p^{g}-\bar{p}^{g},\qquad V=U-I,\qquad d=d-0. $$

Then we have the stratified VRT problem in a perturbation form:

1.4 $$\left\{ \begin{array}{cc} \rho v_{t}+\rho v\cdot \nabla v+\mathrm{div}S(\sigma ,v,V+I)=0 & \text{in }\Omega \setminus \Sigma (t),\\ V_{t}+v\cdot \nabla V=\nabla v(V+I) & \text{in }\Omega \setminus \Sigma (t),\\ \mathrm{div}v=0 & \text{in }\Omega \setminus \Sigma (t),\\ d_{t}+v_{1}\partial_{1}d+v_{2}\partial_{2}d=v_{3} & \text{on }T,\\ 〚v〛=0,\;〚S(\sigma ,v,(V+I))-g\rho dI〛\nu =0 & \text{on }\Sigma (t),\\ v=0 & \text{on }\Sigma_{-}^{+},\\ d{|}_{t=0}=d_{0} & \text{on }\Sigma (0),\\ v_{\pm }{|}_{t=0}=v_{0},\;V_{\pm }{|}_{t=0}=V_{0} & \text{in }\Omega \setminus \Sigma (0), \end{array}\right.$$

where $\Sigma^{+}_{-}:=\Sigma_{-}\cup\Sigma_{+}$, $\mathcal {S}(\sigma,v,V+I)$ is defined by (1.3) with $(\sigma,v,V+I)$ in place of $(p_{\pm},v_{\pm},U_{\pm})$, and we omit the subscript ± in the jump notation 〚⋅〛 for simplicity. The equilibrium-state solution of (1.4) is $(v,V,d,\sigma)=(0,0,0,0)$.

The movement of the free interface $\Sigma(t)$ and the subsequent change of the domains $\Omega_{\pm}(t)$ in Eulerian coordinates result in severe mathematical difficulties. To circumvent such difficulties, we switch our analysis to Lagrangian coordinates, so that the interface and the domains stay fixed in time. To this end, we define the fixed Lagrangian domains $\Omega_{+}=\mathbb{T}\times(0,m)$ and $\Omega_{-}=\mathbb {T}\times(-l,0)$ and assume that there exist invertible mappings

$$ \zeta^{0}_{\pm}:\Omega_{\pm}\rightarrow \Omega_{\pm}(0) $$

such that

1.5 $$ \begin{aligned} \Sigma(0)=\zeta^{0}_{\pm}( \Sigma),\qquad \Sigma_{+}=\zeta^{0}_{+}( \Sigma_{+}),\qquad \Sigma_{-}=\zeta^{0}_{-}( \Sigma_{-}), \end{aligned} $$

and

1.6 $$ \begin{aligned} \det(\nabla\zeta_{0})=1. \end{aligned} $$

The first condition in (1.5) means that the initial interface $\Sigma(0)$ is parameterized by the mapping $\zeta^{0}_{\pm}$ restricted to Σ, whereas the latter two conditions in (1.5) mean that $\zeta^{0}_{\pm}$ map the fixed upper and lower boundaries into themselves. Define the flow maps $\zeta_{\pm}$ as the solutions to

1.7 $$ \textstyle\begin{cases} \partial_{t}\zeta_{\pm}(y,t)=v_{\pm}(\zeta_{\pm}(y,t),t)&\mbox{in }\Omega_{\pm}, \\ \zeta_{\pm}(y,0)=\zeta^{0}_{\pm}(y)&\mbox{in }\Omega_{\pm}. \end{cases} $$

We denote the Eulerian coordinates by $(x,t)$ with $x=\zeta(y,t)$, whereas the fixed $(y,t)\in\Omega\times\mathbb{R}^{+}$stand for the Lagrangian coordinates. Here we have denoted $\Omega_{+}\cup\Omega _{-}$ by Ω.

To switch back and forth from Lagrangian to Eulerian coordinates, we assume that $\zeta_{\pm}(\cdot,t)$ are invertible and $\Omega_{\pm }(t)=\zeta_{\pm}(\Omega_{\pm},t)$, and since $v_{\pm}$ and $\zeta ^{0}_{\pm}$ are all continuous across Σ, we have $\Sigma (t)=\zeta_{\pm}(\Sigma,t)$. In other words, the Eulerian domains of upper and lower fluids are the images of $\Omega_{\pm}$ under the mappings $\zeta_{\pm}$, and the free interface is the image of Σ under the mappings $\zeta_{\pm}(\cdot,t)$. In view of the nonslip boundary condition $v_{\pm}|_{\Sigma_{\pm}}=0$, we have

$$ y=\zeta_{\pm}(y,t)\quad \mbox{on }\Sigma_{\pm}. $$

Moreover, by the incompressible condition (1.1)_3_ we have

1.8 $$ \begin{aligned} \det(\nabla\zeta_{\pm})=1\quad \mbox{in }\Omega_{\pm} \end{aligned} $$

as well as the initial condition (1.6), see [19, Proposition 1.4] for the derivation.

In Lagrangian coordinates the deformation tensor $\tilde{U}(y,t)$ is defined by a Jacobi matrix of $\zeta_{\pm}(y,t)$:

$$ \tilde{U}(y,t):=\nabla\zeta_{\pm}(y,t),\quad\mbox{i.e., }\tilde {U}_{ij}:=\partial_{j}\bigl(\zeta_{\pm}(y,t) \bigr)_{i}. $$

Here and in what follows, $\partial_{j}$ denotes the partial derivative with respect to the *j*th component of the spatial variables. When we study this deformation tensor in Eulerian coordinates, we denote it by $U_{\pm}(x,t):=\tilde{U}(\zeta ^{-1}_{\pm}(x,t),t)$. Applying the chain rule, it is easy to see that $U_{\pm}(x,t)$ satisfies the transport equation

$$ \partial_{t}U_{\pm}+v_{\pm}\cdot\nabla U_{\pm}=\nabla v_{\pm }\cdot\nabla U_{\pm}\quad \mbox{in }\Omega_{\pm}(t). $$

Now, setting

1.9 $$ \zeta=\chi_{+}\zeta_{+}+\chi_{-} \zeta_{-},\qquad \eta=\zeta-y $$

and the Lagrangian unknowns

$$ (u,q) (y,t)=(v,\sigma) \bigl(\zeta(y,t),t\bigr)\quad \mbox{for }(y,t)\in\Omega \times\mathbb{R}^{+}, $$

we see that in Lagrangian coordinates the evolution equations for *u* and *q* read as

1.10 $$\left\{ \begin{array}{cc} \eta_{t}=u & \text{in }\Omega ,\\ \rho u_{t}+{\mathrm{div}}_{A}S_{A}(q,u,\nabla \eta +I)=0 & \text{in }\Omega ,\\ {\mathrm{div}}_{A}u=0 & \text{in }\Omega ,\\ 〚u〛=〚\eta 〛=0,\;〚S_{A}(q,u,\nabla \eta +I)-g\rho \eta_{3}I〛\vec{n}=0 & \text{on }\Sigma ,\\ u=0,\;\eta =0 & \text{on }\Sigma_{-}^{+},\\ u{|}_{t=0}=u_{0},\;\eta {|}_{t=0}=\eta_{0} & \text{in }\Omega , \end{array}\right.$$

where we have denoted

1.11 $$\begin{aligned} &\mathcal{S}_{\mathcal{A}}(q,u,\nabla\eta+I):=qI-\mu\mathcal {S}_{\mathcal{A}}(u)-\kappa\rho\bigl(S(\eta)+\nabla\eta\nabla\eta ^{T}\bigr), \\ & \mathcal{S}_{\mathcal{A}}(u):=\nabla_{\mathcal{A}}u+\nabla _{\mathcal{A}}u^{T}, \end{aligned}$$

1.12 $$\begin{aligned} & \vec{n}:=\frac{\partial_{1}(\eta+y)\times\partial _{2}(\eta+y)}{ \vert \partial_{1}(\eta+y)\times\partial_{2}(\eta +y) \vert }|_{\Sigma}= \frac{\mathcal{A}e_{3}}{ \vert \mathcal {A}e_{3} \vert }|_{\Sigma} \end{aligned}$$

for the unit normal to $\Sigma(t)=\zeta(\Sigma,t)$. Noting that 〚η〛=0 on Σ, we have

$$〚\partial_{i}\eta 〛=0\text{ on }\Sigma \;\text{for }i=1\text{ and }2.$$

Thus the definition of *n⃗* in (1.12) is reasonable. In what follows, we call problem (1.10) the transformed stratified VRT problem. Next, we further introduce the notations involving $\mathcal{A}$. The matrix $\mathcal{A}:=(\mathcal{A}_{ij})_{3\times3}$ via $\mathcal {A}^{T}=(\nabla\zeta)^{-1}:=(\partial_{j}\zeta_{i})^{-1}_{3\times 3}$, and the differential operator $\nabla_{\mathcal{A}}$ is defined by

$$ \nabla_{\mathcal{A}}w:=(\nabla_{\mathcal{A}}w_{1}, \nabla_{\mathcal {A}}w_{2},\nabla_{\mathcal{A}}w_{3})^{T} \quad \mbox{and}\quad \nabla _{\mathcal{A}}w_{i}:=( \mathcal{A}_{1k}\partial_{k}w_{i},\mathcal {A}_{2k}\partial_{k}w_{i},\mathcal{A}_{3k} \partial_{k}w_{i})^{T} $$

for vector functions $w:=(w_{1},w_{2},w_{3})$, and the differential operator $\mathrm{div}_{\mathcal{A}}$ is defined by

1.13 $$ \begin{aligned} \mathrm{div}_{\mathcal{A}}(f_{1},f_{2},f_{3})= (\mathrm{div}_{\mathcal{A}}f_{1}, \mathrm{div}_{\mathcal{A}}f_{2}, \mathrm{div}_{\mathcal{A}}f_{3})^{T}\quad \mbox{and}\quad \mathrm{div}_{\mathcal{A}}f_{i}:=\mathcal{A}_{lk} \partial_{k}f_{il} \end{aligned} $$

for vector functions $f_{i}:=(f_{i1},f_{i2},f_{i3})^{T}$. It should be noted that we have used the Einstein convention of summation over repeated indices. In addition, we define $\Delta_{\mathcal{A}}X:=\mathrm{div}_{\mathcal{A}} \nabla_{\mathcal{A}}X$.

Finally, we introduce some properties of $\mathcal{A}$. In view of the definition of $\mathcal{A}$ and (1.8), we see that

1.14 $$ \begin{aligned} \mathcal{A}=\bigl(\mathcal{A}^{\ast}_{ij} \bigr)_{3\times3}, \end{aligned} $$

where $\mathcal{A}^{\ast}_{ij}$ is the algebraic complement minor of the $(i,j)$th entry of the matrix $(\partial_{j}\zeta_{i})_{3\times 3}$. In addition, we have

1.15 $$ \mathcal{A}_{ji}\partial_{l} \zeta_{j}=\mathcal{A}_{ij}\partial_{j}\zeta _{l}=\delta_{il} $$

and

1.16 $$ \begin{aligned} \partial_{k} \mathcal{A}^{\ast}_{ik}=0\quad \mbox{or}\quad \partial _{k}\mathcal{A}_{ik}=0, \end{aligned} $$

where $\delta_{il}=1$ for $i=l$ and $\delta_{il}=0$ for $i\neq l$.

If $(u,\eta)$ is very small, then the small terms of second order (i.e., the nonlinear terms) in (1.10) can be neglected, and we thus obtain the following linearized stratified VRT problem:

1.17 $$\left\{ \begin{array}{cc} \eta_{t}=u & \text{in }\Omega ,\\ \rho u_{t}+\nabla q=\mu \Delta u+\kappa \rho \mathrm{div}S(\eta ) & \text{in }\Omega ,\\ \mathrm{div}u=0 & \text{in }\Omega ,\\ 〚u〛=〚\eta 〛=0,\;〚(q-g\rho \eta_{3})I-S(\mu u+\kappa \rho \eta )〛e_{3}=0 & \text{on }\Sigma ,\\ u=0,\;\eta =0 & \text{on }\Sigma_{-}^{+},\\ u{|}_{t=0}=u_{0},\;\eta_{t=0}=\eta_{0} & \text{in }\Omega , \end{array}\right.$$

where $S(f)=\nabla f+\nabla f^{T}$ for $f=\eta$, and $\mu u+\kappa \rho\eta$. The linearized problem is convenient to analyze in order to have an insight into the physical and mathematical mechanisms of the stratified VRT problem. In fact, using a standard energy method, Jiang et al. [17] found stability and instability criteria of the above linearized stratified VRT problem.

Before recalling the stability and instability criteria, we introduce some simplified notations:
 where *k* is a nonnegative integer, *s* is a real number, and the positive constant *c* may depend on the domain occupied by the fluids and other known physical parameters such as *ρ*, *μ*, *g*, and *κ* and varies from line to line.

Next, we introduce the stability and instability criteria of (1.17). We consider normal mode solutions of (1.17) in the form

$$ u(y,t)=\tilde{u}(y)e^{\Lambda t},\qquad \eta(y,t)=\tilde{ \eta}(y)e^{\Lambda t},\qquad q(y,t)=\tilde{q}(y)e^{\Lambda t}\quad \mbox{for some constant } \Lambda>0. $$

Substituting this ansatz into (1.17), we obtain the eigenvalue problem

1.18 $$\left\{ \begin{array}{cc} \Lambda \tilde{\eta }=\tilde{u} & \text{in }\Omega ,\\ \Lambda \rho \tilde{u}+\nabla \tilde{q}=\mu \Delta \tilde{u}+\kappa \rho \mathrm{div}S(\tilde{\eta }) & \text{in }\Omega ,\\ \mathrm{div}\tilde{u}=0 & \text{in }\Omega ,\\ 〚\tilde{u}〛=0,\;〚(\tilde{q}-g\rho {\tilde{\eta }}_{3})I-S(\mu \tilde{u}+\kappa \rho \tilde{\eta })〛e_{3}=0 & \text{on }\Sigma ,\\ \tilde{u}=0,\;\tilde{\eta }=0 & \text{on }\Sigma_{-}^{+}. \end{array}\right.$$

Eliminating *η̃* by using the first equation, we arrive at the boundary value problem

1.19 $$\left\{ \begin{array}{cc} \Lambda^{2}\rho \tilde{u}+\Lambda \nabla \tilde{q}-\mathrm{div}S((\Lambda \mu +\kappa \rho )\tilde{u})=0 & \text{in }\Omega ,\\ \mathrm{div}\tilde{u}=0 & \text{in }\Omega ,\\ 〚\tilde{u}〛=0,\;〚(\Lambda \tilde{q}-g\rho {\tilde{u}}_{3})I-S((\Lambda \mu +\kappa \rho )\tilde{u})〛e_{3}=0 & \text{on }\Sigma ,\\ \tilde{u}=0,\;\tilde{\eta }=0 & \text{on }\Sigma_{-}^{+}. \end{array}\right.$$

Multiplying (1.19)_1_ by *ũ* in $L^{2}$ and using the formula of integral by parts and conditions (1.19)_2_–(1.19)_4_, we have

$$ \Lambda^{2} \Vert \sqrt{\rho}\tilde{u} \Vert ^{2}_{0}= \tilde{E}(\tilde{u}) - \bigl\Vert \sqrt{\Lambda\mu}S(\tilde{u}) \bigr\Vert ^{2}_{0}/2 $$

with

$$\tilde{E}(\tilde{u}):=g〚\rho 〛|{\tilde{u}}_{3}{|}_{0}^{2}-{∥\sqrt{\kappa \rho }S(\tilde{u})∥}_{0}^{2}/2.$$

By the classical theory of linear RT instability, if
 then the linearized stratified VRT problem is unstable. Obviously, the above condition is equivalent to

1.20 $$ C_{\kappa}>1, $$

where we have defined

$$C_{\kappa }:=\underset{w\in H_{\sigma }^{1}}{\sup}\frac{2g〚\rho 〛|w_{3}{|}_{0}^{2}}{{∥\sqrt{\kappa \rho }S(w)∥}_{0}^{2}}.$$

Jiang et al. [17] used the discrete Fourier transformation to prove that there exist unstable solutions to the linearized stratified VRT problem under $C_{\kappa}>1$. Moreover, they further verified that the transformed stratified VRT problem is stable for $C_{\kappa}<1$. The nonlinear stability result shows that the elasticity can inhibit the development of RT instability.

In this paper, we assume that *κ* is a constant. Then the instability criterion (1.20) reduces to

1.21 $$\begin{array}{r} \kappa <\kappa_{c}:=\underset{\omega \in H_{\sigma }^{1}}{\sup}\frac{2g〚\rho 〛|w_{3}{|}_{0}^{2}}{{∥\sqrt{\rho }S(w)∥}_{0}^{2}}>0. \end{array}$$

Under (1.21), we prove that there exists an unstable solution of the linearized stratified VRT problem with a largest growth rate, and the largest growth rate decreases from a positive constant to 0 as *κ* increases from 0 to $\kappa _{c}$. Next, we introduce the definition of largest growth rate.

### Definition 1.1

We call $\Lambda>0$ the largest growth rate of RT instability in the linearized stratified VRT problem if it satisfies the following two conditions:

1. For any classical solution $(u,\eta)$ of the linearized stratified VRT problem with an associated pressure *q*, we have, for any $t\geq0$,
  1.22 $$\begin{aligned} & \bigl\Vert u(t) \bigr\Vert _{1}^{2}+ \Vert u_{t} \Vert ^{2}_{0 }+ \int_{0}^{t} \bigl\Vert u(s) \bigr\Vert ^{2}_{1}\,ds\lesssim e^{2\Lambda t}\bigl( \Vert u_{0} \Vert _{1}^{2} +I_{0}\bigr), \end{aligned}$$
  1.23 $$\begin{aligned} & \bigl\Vert \eta(t) \bigr\Vert _{1} \lesssim e^{\Lambda t}\bigl( \bigl\Vert (u_{0}, \eta_{0}) \bigr\Vert _{1}+\sqrt{I_{0}}\bigr), \end{aligned}$$
  where $I_{0}:=\|\sqrt{\rho}u_{t}(0)\|^{2}_{0} -\tilde{E}(u_{0})$.
2. There exists a solution $(u,\eta,q)$ of the linearized stratified VRT problem in the form
  $$(u,\eta,q):=e^{\Lambda t}(\tilde{u},\tilde{\eta},\tilde{q}), $$
  where $(\tilde{u},\tilde{\eta},\tilde{q})\in H^{2}\times H^{2}\times H^{1}$.

Now we state our result on the linearized stratified VRT problem.

### Theorem 1.1

*Assume that* $\mu>0$, $\rho>0$, $g>0$, *and* 〚ρ〛>0. *If the constant* $\kappa\in[0,\kappa_{c})$, *then there exists a largest growth rate* (*see Definition* 1.1) $\Lambda>0$ *such that there is an unstable solution to the linearized stratified VRT problem* (1.17) *of the form*

$$(u,\eta,q):=e^{\Lambda t}(\tilde{u},\tilde{u}/\Lambda,\tilde{q}), $$

*where* $(\tilde{u},\tilde{q})\in(H^{1}_{\sigma}\cap H^{\infty})\times H^{\infty}$ *solves the boundary value problem* (1.19). *Moreover*, $\|\tilde{u}_{3}\|_{0}\|(\tilde{u}_{1}, \tilde{u}_{2})\|_{0}|\tilde{u}_{3}|_{0}>0$.

*In addition*, *for given* *g*, *ρ*, *and* *μ*, *we can regard* $\Lambda _{\kappa}:=\Lambda$ *as a function of* $\kappa\in[0,\kappa_{c})$. *It enjoys the following properties*:

1.24 $$ \Lambda_{\kappa}\textit{ strictly decreases and is continuous with respect to }\kappa, $$

*and*

1.25 $$ \Lambda_{\kappa}\leq m:=\max \biggl\{ \frac{\rho_{+}(\kappa _{c}-\kappa)}{\mu_{+}}, \frac{\rho_{-}(\kappa_{c}-\kappa)}{\mu _{-}} \biggr\} . $$

*In particular*, *we have* $\Lambda_{\kappa}\rightarrow0$ *as* $\kappa \rightarrow\kappa_{c}$.

The proof of Theorem 1.1 is based on the modified variational method [5, 9, 13, 14]. Next, we briefly introduce the proof of Theorem 1.1. Obviously, the linearized stratified VRT problem (1.17) is unstable if there exists a solution $(\tilde{u},\tilde{q},\Lambda)$ to the boundary-value problem (1.19) with $\Lambda>0$. In view of the basic idea of the modified variational method, to look for the solution $(\tilde{u},\tilde{q},\Lambda)$, we will use a modified variational approach and thus modify (1.19) as follows:

1.26 $$\left\{ \begin{array}{cc} \alpha (s)\rho \tilde{u}+s\nabla \tilde{q}-\mathrm{div}S((s\mu +\kappa \rho )\tilde{u})=0 & \text{in }\Omega ,\\ \mathrm{div}\tilde{u}=0 & \text{in }\Omega ,\\ 〚\tilde{u}〛=0,\;〚(s\tilde{q}-g\rho {\tilde{u}}_{3})I-S((s\mu +\kappa \rho )\tilde{u})〛e_{3}=0 & \text{on }\Sigma ,\\ \tilde{u}=0,\;\tilde{\eta }=0 & \text{on }\Sigma_{-}^{+}, \end{array}\right.$$

where $s>0$ is a given parameter, and $\alpha(s)$ depends on *s*.

Multiplying (1.26)_1_ by *ũ* and integrating the resulting identity, we get

$$ \alpha(s) \Vert \sqrt{\rho}\tilde{u} \Vert _{0}^{2} = \tilde{E}(\tilde{u}) -s \Phi(\tilde{u}), $$

where

$$\tilde{E}(\tilde{u}):=g〚\rho 〛|{\tilde{u}}_{3}{|}_{0}^{2}-{∥\sqrt{\kappa \rho }S(\tilde{u})∥}_{0}^{2}/2,\;\Phi (\tilde{u})={∥\sqrt{\mu }S(\tilde{u})∥}_{0}^{2}/2.$$

Let $\mathcal{E}(\tilde{u},s):= \tilde{E}(\tilde{u}) -s \Phi(\tilde{u})$. Then $\mathcal{E}(\tilde{u},s)$ is the energy functional of (1.26) (sometimes, we denote $\mathcal{E}(\tilde{u},s)$ by $\mathcal{E}(\tilde{u})$ for simplicity). Thus, for any given $s>0$, by a standard variational approach there exists a maximizer $\tilde{u}\in\mathcal{A}$ of the energy functional $\mathcal{E}(\tilde{u},s)$. Moreover, *ũ* is just the weak solution to (1.26), and $\alpha(s)$ in (1.26) is defined by the relation

1.27 $$ \alpha(s)=\sup_{{\tilde{u}}\in\mathcal{A}}\mathcal{E}(\tilde{u},s) \in\mathbb{R}. $$

Exploiting the classical regularity theory on the stratified steady Stokes problem, *ũ* indeed is a classical solution of the boundary-value problem (1.26) with an associated function *q̃* and an associated constant $\alpha(s)>0$ defined by (1.27); see Lemma 2.1 for detailed results.

In view of the definition of $\alpha(s)$, we can infer that the function $\alpha(s )$ enjoys the three properties: $\alpha(s )\in C_{\mathrm{loc}}^{0,1}(0,\infty)$, $\alpha(s )$ is strictly decreasing, and $\lim_{s\rightarrow0}\alpha(s )>0$. Obviously, $\sqrt{\alpha(s )}$ also possesses these properties and thus has a fixed point on some internal $(0,\mathcal{G})$, that is, there exists Λ satisfying the fixed-point relation

1.28 $$ \Lambda=\sqrt{\alpha(\Lambda)} \in(0,\mathcal{G}), $$

which immediately implies that there exists a nontrivial solution $\tilde{u}\in H^{2} $ to (1.19) with a positive constant Λ defined by (1.28), and therefore the linear instability follows; see Proposition 2.1 for details. Moreover, Λ is the largest growth rate of RT instability in the linearized stratified VRT problem; see Proposition 2.2. To emphasize the dependence of $\alpha(s)$, Λ, and $\mathcal {G}$ upon *κ*, we denote them by $\alpha(s,\kappa)$, $\Lambda _{\kappa}$, and $\mathcal{G}_{\kappa}$, respectively.

In addition, we can further prove that, for fixed *s*, $\alpha (s,\kappa)$ strictly decreases and is continuous with respect to *κ*; moreover, there exists an estimate $\mathcal{G}_{\kappa}\leq m$; see Lemma 2.4. Consequently, using the fixed-point relation (1.28), Lemma 2.4, and the definition of continuity, we can show that $\Lambda_{\kappa}$ also inherits the properties of $\alpha(s,\kappa)$ with respect to *κ* and thus get properties (1.24)–(1.25) of $\Lambda _{\kappa}$. In the next section, we provide a detailed proof of Theorem 1.1.

## Proof of Theorem 1.1

To begin with, we prove that a maximizer of (1.27) exists and that the corresponding Euler–Lagrange equations are equivalent to (1.26).

### Lemma 2.1

*Under the assumptions of Theorem* 1.1, *for any but fixed* $s>0$, *the following assertions are valid*.

1. *In the variational problem* (1.27), $\mathcal{E}(\tilde {u})$ *achieves its supremum on* $\mathcal{A}$.
2. *Let* $\tilde{u}_{0}$ *be a maximizer*, *and let* $\alpha:=\sqrt{\sup_{\tilde{u}\in\mathcal{A}}\mathcal{E}(\tilde{u})}$. *Then there exists a pressure* $\tilde{q}_{0}$ *associated with* $\tilde{u}_{0}$ *such that the triple* $(\tilde{u}_{0},\tilde{q}_{0},\alpha)$ *satisfies the boundary problem* (1.26). *Moreover*, $(\tilde {u}_{0},\tilde{q}_{0})\in(H^{1}_{\sigma}\cap H^{\infty})\cap H^{\infty}$.

### Proof

(1) Noting the estimate

2.1 $$ \vert w \vert _{0}^{2}\leq2 \Vert w \Vert _{0} \Vert w \Vert _{1}\quad\mbox{for any }w\in H_{0}^{1}, $$

we can use the Cauchy–Schwarz and Korn inequalities

$$\Vert w \Vert _{1}\lesssim \bigl\Vert S(w) \bigr\Vert _{0}\quad \mbox{for }w\in H_{0}^{1} $$

to see that $\alpha(s)$ has an upper bound for any $\tilde{u}\in \mathcal{A}$. Hence $\alpha(s)$ has a maximizing sequence. Let $\tilde{u}_{n}\in\mathcal{A}$ be a maximizing sequence. Then $\{ \mathcal{E}(\tilde{u}_{n})\}_{n=1}^{\infty}$ has a lower bound. Thus there exists a constant *c* such that

$$c+{∥\sqrt{\kappa \rho }S({\tilde{u}}_{n})∥}_{0}^{2}/2+s\Phi ({\tilde{u}}_{n})\leq g〚\rho 〛|{\tilde{u}}_{n3}{|}_{0}^{2}.$$

Using (2.1) and the Cauchy–Schwarz and Korn inequalities, we can deduce from this estimate that $\tilde{u}_{n}$ is bounded in $H^{1}$. So, there exist $\tilde{u}_{0}\in H^{1}\cap\mathcal{A}$ and a subsequence (still denoted by $\tilde{u}_{n}$ for simplicity) such that $\tilde{u}_{n}\rightarrow\tilde{u}_{0}$ weakly in $H^{1}$ and strongly in $L^{2}$. Further, we have $\tilde{u}_{n}\rightarrow\tilde {u}_{0}$ strongly in $L^{2}(\Sigma)$. Indeed, in view of (2.1),

$$\vert \tilde{u}_{n}-\tilde{u}_{0} \vert _{0}^{2} \leq2 \Vert \tilde{u}_{n}- \tilde{u}_{0} \Vert _{0} \Vert \tilde{u}_{n}- \tilde{u}_{0} \Vert _{1} \lesssim \Vert \tilde{u}_{n}-\tilde{u}_{0} \Vert _{0} \rightarrow0\quad \mbox{as } n \rightarrow\infty. $$

Therefore, by the lower semicontinuity we have

$$\begin{array}{rl} \underset{\tilde{u}\in A}{\sup}E(\tilde{u}) & =\underset{n\to \infty }{lim sup}E({\tilde{u}}_{n})\\ & =\lim_{n\to \infty }g〚\rho 〛|{\tilde{u}}_{n3}{|}_{0}^{2}+\frac{1}{2}\underset{n\to \infty }{lim sup}\left(-{∥\sqrt{\kappa \rho +s\mu }S({\tilde{u}}_{n})∥}_{0}^{2}\right)\\ & =\lim_{n\to \infty }g〚\rho 〛|{\tilde{u}}_{n3}{|}_{0}^{2}-\frac{1}{2}\underset{n\to \infty }{lim inf}{∥\sqrt{\kappa \rho +s\mu }S({\tilde{u}}_{n})∥}_{0}^{2}\\ & \leq g〚\rho 〛|{\tilde{u}}_{0}{|}_{0}^{2}-\frac{1}{2}{∥\sqrt{\kappa \rho +s\mu }S({\tilde{u}}_{0})∥}_{0}^{2}\\ & =E({\tilde{u}}_{0})\leq \underset{\tilde{u}\in A}{\sup}E(\tilde{u}), \end{array}$$

which shows that $\mathcal{E}(\tilde{u})$ achieves its supremum on $\mathcal{A}$.

(2) To show the second assertion, we notice that since $\mathcal {E}(\tilde{u})$ and $J(\tilde{u})$ are homogeneous of degree 2, (1.27) is equivalent to

2.2 $$ \alpha=\sup_{w\in H_{\sigma}^{1}}\frac{\mathcal{E}(w)}{J(w)}. $$

For any given $\tau\in\mathbb{R}$ and ${w}\in H_{\sigma}^{1}$, we take $\tilde{ {w}}:=\tilde{{u}}_{0}+\tau{w}$. Then (2.2) implies

$$ \mathcal{E}(\tilde{ {w}})-\alpha J(\tilde{ {w}})\leq0. $$

If we set $I(\tau)=\mathcal{E}(\tilde{ {w}})-\alpha J(\tilde{ {w}})$, then we see that $I(\tau)\in C^{1}(\mathbb{R})$, $I(\tau )\leq0$ for all $\tau\in\mathbb{R}$, and $I(0)=0$. This implies $I'(0)=0$. Hence a direct computation leads to

$$\alpha \int \rho {\tilde{u}}_{0}\cdot w\;dy+\frac{1}{2}\int (\kappa \rho +s\mu )S({\tilde{u}}_{0}):S(w)\;dy=g〚\rho 〛\int_{\Sigma }{\tilde{u}}_{03}w_{3}\;dy_{h},$$

or

$$\alpha \int \rho {\tilde{u}}_{0}\cdot w\;dy+\int (\kappa \rho +s\mu )S({\tilde{u}}_{0}):\nabla w\;dy=g〚\rho 〛\int_{\Sigma }{\tilde{u}}_{03}w_{3}\;dy_{h},$$

which implies that $\tilde{u}_{0}\in H^{1}$ is a weak solution to the boundary problem (1.26). Here $\tilde{u}_{03}$ denotes the third component of $\tilde{u}_{0}$.

Now we consider the following stratified steady Stokes problem:

2.3 $$\left\{ \begin{array}{cc} -(s\mu +\kappa \rho )\Delta \tilde{u}+s\nabla \tilde{q}=H & \text{in }\Omega ,\\ \mathrm{div}\tilde{u}=0 & \text{in }\Omega ,\\ 〚\tilde{u}〛=0,\;〚s\tilde{q}I-(s\mu +\kappa \rho )S(\tilde{u})〛e_{3}=F & \text{on }\Sigma ,\\ \tilde{u}=0,\;\tilde{\eta }=0 & \text{on }\Sigma_{-}^{+}. \end{array}\right.$$

It follows from the classical regularity of stratified steady Stokes problem in [24, Lemma A.8] that if $\mathcal{H}\in H^{m}$ and $\mathcal{F}\in H^{m+1/2}$ for $m\geq0$, then there exists a unique solution $(\tilde{u}, \tilde{q})\in H^{m+2} \times H^{m+1}$ to (2.3) such that

$$\begin{array}{rl} {∥\tilde{u}∥}_{m+2}+{∥\nabla \tilde{q}∥}_{m}+|〚\tilde{q}〛{|}_{m+1/2} & ≲{∥H∥}_{m}+|F{|}_{m+1/2}. \end{array}$$

We suppose that $\mathcal{H}=-\alpha(s)\rho\tilde{u}_{0}\in H^{1}$ and $F=g〚\rho 〛{\tilde{u}}_{03}e_{3}\in H^{1/2}$. Then by the above regularity result there exists a solution $(\tilde {u},\tilde{q})\in H^{2}\times H^{1}$ to (2.3). Since $\tilde{u}_{0}$ is a weak solution to (2.3), we can easily check that $\tilde{u}_{0}=\tilde{u}$. Thus by a standard bootstrap method of improving the regularity we can easily see that $(\tilde{u}_{0},\tilde{q}_{0})\in(H^{1}_{\sigma}\cap H^{\infty})\times H^{\infty}$. This completes the proof. □

To prove that there is a fixed point Λ such that $\Lambda =\sqrt{\alpha(\Lambda)}>0$, we further give some properties of $\alpha(s)$ with respect to $s>0$.

### Lemma 2.2

*Under the assumptions of Theorem* 1.1, *the function* $\alpha(s)$ *defined on* $(0,\infty)$ *enjoys the following properties*:

1. $\alpha(s)\in C^{0,1}_{\mathrm{loc}}(0,\infty)$ *is strictly decreasing in the variable* *s*.
2. *There are constants* $c_{1}$, $c_{2}>0$, *which depend on* *g*, *ρ*, *κ*, *and* *μ*, *such that*
  2.4 $$ \alpha(s)\geq c_{1}-c_{2} s. $$

### Proof

(1) Let $s_{2}>s_{1}$. By Proposition 2.1 there exists a function $\tilde{u}^{s_{i}}\in\mathcal{A}\cap H^{\infty}$ such that $\tilde{u}^{s_{i}}\neq0$ and

$$ \alpha(s_{i})=\tilde{E}\bigl(\tilde{u}^{s_{i}}\bigr) -s_{i}\Phi\bigl(\tilde{u}^{s_{i}}\bigr),\quad i=1,2. $$

Using Korn’s inequality, we have

$$ 0< \bigl\Vert \tilde{u}^{s_{i}} \bigr\Vert _{{1}}^{2} \lesssim \bigl\Vert \Phi\bigl(\tilde{u}^{s_{i}}\bigr) \bigr\Vert _{0}^{2}, $$

and thus

$$ \alpha(s_{2})\leq\alpha(s_{1})+(s_{1}-s_{2}) \Phi\bigl(\tilde{u}^{s_{2}}\bigr)< \alpha(s_{1}). $$

Hence $\alpha(s)$ is strictly decreasing on $(0,\infty)$.

Now we turn to show $\alpha(s)\in C^{0,1}_{{\rm loc}}(0,\infty)$. Let $[b_{1},b_{2}]\subset(0,\infty)$ be a bounded interval. Then, for any $s\in[b_{1},b_{2}]$, there exists a function $\tilde{u}^{s}$ satisfying $\alpha(s)=E(\tilde{u}^{s})-s\Phi(\tilde{u}^{s})$. Thus, in view of the monotonicity of $\alpha(s)$, we have that

$$ \alpha(b_{2})+b_{1}\Phi\bigl(\tilde{u}^{s}\bigr)/2 \leq E\bigl(\tilde{u}^{s}\bigr)-(s/2)\Phi \bigl(\tilde{u}^{s} \bigr)\leq \alpha(s/2)\leq\alpha(b_{1}/2), $$

which yields

$$\Phi\bigl(\tilde{u}^{s}\bigr)\leq2 \bigl(\alpha(b_{1}/2)- \alpha(b_{2}) \bigr)/{b_{1}}:=K \quad \mbox{for any } s \in[b_{1},b_{2}]. $$

Thus, for any $s_{1},s_{2}\in[b_{1},b_{2}]$,

$$\alpha(s_{1})-\alpha(s_{2})\leq E\bigl(\tilde{u}^{s_{1}} \bigr)-s_{1}\Phi\bigl(\tilde{u}^{s_{1}}\bigr) - \bigl(E\bigl( \tilde{u}^{s_{1}}\bigr)-s_{2}\Phi\bigl(\tilde{u}^{s_{1}} \bigr) \bigr)\leq K \vert s_{2}-s_{1} \vert . $$

Reversing the role of indices 1 and 2 in the derivation of this inequality, we obtain the same boundedness with the indices switched. Therefore, we deduce that

$$ \begin{aligned} \bigl\vert \alpha(s_{1})- \alpha(s_{2}) \bigr\vert \leq K \vert s_{1}-s_{2} \vert , \end{aligned} $$

which yields $\alpha(s)\in C^{0,1}_{\mathrm{loc}}(0,\infty)$.

(2) By the instability condition $\kappa<\kappa_{{c}}$ of the linearized stratified VRT problem there exists a function $\tilde{u}\in H_{\sigma}^{1}$ such that

$$\begin{array}{r} g〚\rho 〛|{\tilde{u}}_{3}{|}_{0}^{2}-{∥\sqrt{\kappa \rho }S(\tilde{u})∥}_{0}^{2}/2>0. \end{array}$$

Thus we have

$$\begin{array}{rl} \alpha (s) & =\underset{w\in A}{\sup}E(w,s)=\underset{w\in H_{\sigma }^{1}}{\sup}\frac{E(w,s)}{J(w)}\\ & \geq \frac{g〚\rho 〛|{\tilde{u}}_{3}{|}_{0}^{2}-{∥\sqrt{\kappa \rho }S(\tilde{u})∥}_{0}^{2}/2}{\int \rho |\tilde{u}{|}^{2}\;dy}-\frac{s{∥\sqrt{\mu }S(\tilde{u})∥}_{0}^{2}/2}{\int \rho |\tilde{u}{|}^{2}\;dy}:=c_{1}-sc_{2} \end{array}$$

for two positive constants $c_{1}:=c_{1}(g,\rho,\kappa)$ and $c_{2}:=c_{2}(\mu,\rho)$. This completes the proof of Lemma 2.2. □

Next, we prove that there exists a pair of functions $(\tilde {u},\tilde{q})$ satisfying (1.19) with a growth rate Λ by a fixed-point argument.

Let

2.5 $$ \begin{aligned} \mathcal{G}:=\sup\bigl\{ s|\alpha( \tau)>0\mbox{ for any }\tau\in (0,s)\bigr\} . \end{aligned} $$

By Lemma 2.2, $\mathcal{G}>0$; moreover, $\alpha(s)>0$ for any $s<\mathcal{G}$. Since $\alpha(s)=\sup_{\tilde{u}\in\mathcal{A}}\mathcal{E}(\tilde {u},s)<\infty$, using the monotonicity of $\alpha(s)$, we see that

2.6 $$ \begin{aligned} \lim_{s\rightarrow0}\alpha(s) \mbox{ exists, and the limit is a positive constant}. \end{aligned} $$

By (2.1) and Korn’s inequalit there are two constants $c_{3}$ and $c_{4}$, depending on the domain Ω and other known physical parameters, such that

$$g〚\rho 〛|{\tilde{u}}_{3}{|}_{0}^{2}\leq c_{3}{∥\tilde{u}∥}_{1}^{2}\;\text{for any }\tilde{u}\in A$$

and

$$ \Vert \tilde{u} \Vert _{1}^{2} \leq c_{4} \bigl\Vert S(\tilde{u}) \bigr\Vert _{0}^{2}/2 \quad\mbox{for any }\tilde{u}\in H^{1}_{0}. $$

Thus, if $s>(c_{3}c_{4}-\kappa\min\{\rho_{-},\rho_{+}\})/\min\{\mu_{-},\mu _{+}\}$, then

$$\begin{array}{rl} & g〚\rho 〛|{\tilde{u}}_{3}{|}_{0}^{2}-\frac{1}{2}{∥\sqrt{\kappa \rho +s\mu }S(\tilde{u})∥}_{0}^{2}\\ & \;\leq \left(c_{3}-\frac{\kappa \min\{\rho_{-},\rho_{+}\}+s\min\{\mu_{-},\mu_{+}\}}{c_{4}}\right){∥\tilde{u}∥}_{1}^{2}<0\;\text{for any }\tilde{u}\in A, \end{array}$$

which implies that

$$ \alpha(s)\leq0\quad\mbox{for any }s> \bigl(c_{3}c_{4}-\kappa\min\{\rho _{-},\rho_{+}\}\bigr)/\min\{ \mu_{-},\mu_{+}\}. $$

Hence $\mathcal{G}<\infty$. Moreover,

2.7 $$ \lim_{s\rightarrow\mathcal{G}} \alpha(s)=0. $$

In view of (2.5)–(2.7) and the continuity of $\alpha(s)$ on $(0, \mathcal{G})$, we can use a fixed-point argument to deduce the following conclusion.

### Lemma 2.3

*Under the assumptions of Theorem* 1.1, *there exists a unique* $\Lambda\in(0,\mathcal{G})$ *such that*

2.8 $$ \Lambda=\sqrt{\alpha(\Lambda)}= \sqrt{\sup_{\tilde{w}\in\mathcal{A}} \mathcal{E}(\tilde{w},\Lambda)}>0. $$

### Proof

We define the function $\varphi:\mathcal{S}=(0,\mathcal {G})\rightarrow(0,\infty)$ by

2.9 $$ \varphi(s)=s/\sqrt{\alpha(s)}. $$

Then $\varphi(s)$ is continuous and strictly increasing with respect to *s*. Moreover, by (2.6) and (2.7) we have $\lim_{s\rightarrow0}\varphi(s)=0$ and $\lim_{s\rightarrow\mathcal {G}}\varphi(s)=+\infty$. Hence there is a unique constant $\Lambda \in(0,\mathcal{G})$ such that $\varphi(\Lambda)=1$, which gives (2.8). □

By Lemma 2.1 there is a solution $(\tilde{u},\tilde {q})\in(H^{1}_{\sigma}\cap H^{\infty})\times H^{\infty}$ to problem (1.19) with Λ constructed in (2.8). Moreover, $\Lambda^{2}=\mathcal{E}(\tilde{u},\Lambda)$, $\tilde{u}_{3}\neq 0$ in Ω, and $\tilde{u}_{3}\neq0$ on Σ by (2.8). Since $\operatorname{div}\tilde{u}=0$ and $\tilde{u}|_{\Sigma ^{+}_{-}}=0$, we immediately get $\tilde{u}^{2}_{1}+\tilde {u}^{2}_{2}\neq0$. Thus we have the following conclusion.

### Proposition 2.1

*Under the assumptions of Theorem* 1.1, *there exists a pair of functions* $(\tilde{u},\tilde{q})\in(H^{1}_{\sigma}\cap H^{\infty})\times H^{\infty}$ *satisfying the boundary problem* (1.19) *with growth rate* $\Lambda>0$ *constructed by* (2.8). *Moreover*, $\|\tilde{u}_{3}\|_{0}\|(\tilde{u}_{1},\tilde{u}_{2})\| _{0}|\tilde{u}_{3}|_{0}>0$.

Obviously, the above conclusion yields the existence of an unstable solution to the linearized stratified VRT problem in Theorem 1.1 in the form

2.10 $$ (u,\eta,q):=e^{\Lambda t}(\tilde{u},\tilde{u}/\Lambda, \tilde{q}), $$

where *ũ* is provided by Proposition 2.1. Moreover, Λ enjoys the following property.

### Proposition 2.2

*Under the assumptions of Theorem* 1.1, *for any classical solution* $(u,\eta)$ *of the linearized stratified VRT problem with an associated pressure* *q*, *for any* $t\geq0$, *we have* (1.22) *and* (1.23), *where* $\Lambda>0$ *is constructed by* (2.8).

### Proof

We use (2.8) and the energy method as in [5] to deduce (1.22)–(1.23) from (1.17). Differentiating (1.17)_2_–(1.17)_5_ with respect to time, we get

2.11 $$\left\{ \begin{array}{cc} \rho u_{tt}+\nabla q_{t}=\mu \Delta u_{t}+\kappa \rho \mathrm{div}S(\eta_{t}) & \text{ in }\Omega ,\\ \mathrm{div}u_{t}=0 & \text{ in }\Omega ,\\ 〚u_{t}〛=0,\;〚(q_{t}-g\rho \partial_{t}\eta_{3})I-S(\mu u_{t}+\kappa \rho \eta_{t})〛e_{3}=0 & \text{ on }\Sigma ,\\ u_{t}=0 & \text{ on }\Sigma_{-}^{+}. \end{array}\right.$$

Multiplying (2.11)_1_ with $u_{t}$, integrating the resulting equation (by parts), and recalling that ${div}u_{t}=0$, we obtain

2.12 $$\begin{array}{rl} & \frac{1}{2}\;\frac{d}{dt}{∥\sqrt{\rho }u_{t}∥}_{0}^{2}+\frac{1}{2}{∥\sqrt{\mu }S(u_{t})∥}_{0}^{2}+\int \kappa \rho S(\eta_{t}):\nabla u_{t}\;dy\\ & \;-g〚\rho 〛\int_{\Sigma }\partial_{t}\eta_{3}\partial_{t}u_{3}\;dy_{h}=0. \end{array}$$

On the other hand, by (1.17)_1_ we have

$$\int_{\Sigma}\partial_{t}\eta_{3} \partial_{t} u_{3}\,{d}y_{h}= \int_{\Sigma }u_{3} \partial_{t} u_{3}\,{d}y_{h}=\frac{1}{2}\,\frac{{d}}{{d}t} \int_{\Sigma}u_{3}^{2} \,{d}y_{h} $$

and

$$\begin{aligned} \int \kappa\rho S(\eta_{t} ) : \nabla u_{t}\,{d}y=& \int \kappa\rho\bigl( \nabla u +\nabla u^{{T}} \bigr):\nabla u_{t}\,{d}y = \frac{ 1 }{4}\,\frac{{d}}{{d}t} \bigl\Vert \sqrt{ \kappa\rho} S(u) \bigr\Vert ^{2}_{0}. \end{aligned} $$

Inserting these equalities into (2.12), we infer that

2.13 $$ \begin{aligned} \frac{{d}}{{d}t} \bigl( \Vert \sqrt{\rho}u_{t} \Vert ^{2}_{0}-\tilde{E}(u) \bigr)+ \bigl\Vert \sqrt{\mu} S(u_{t}) \bigr\Vert ^{2}_{0}=0. \end{aligned} $$

Using the Newton–Leibniz formula and Cauchy–Schwarz inequality, we find that

2.14 $$ \begin{aligned}[b] \Lambda \bigl\Vert \sqrt{\mu}S \bigl(u(t)\bigr) \bigr\Vert _{0}^{2} ={}&\Lambda \bigl\Vert \sqrt{\mu} S( u_{0}) \bigr\Vert _{0}^{2} + 2\Lambda \int_{0}^{t} \int \mu S\bigl(u(s)\bigr):S(u_{s}) \,{d} y\,{d}s \\ \leq{}& \Lambda \bigl\Vert \sqrt{\mu} S( u_{0}) \bigr\Vert _{0}^{2} + \int_{0}^{t} \bigl\Vert \sqrt{\mu } S( u_{s}) \bigr\Vert _{0}^{2} \,{d}\tau\\ &{}+ \Lambda^{2} \int_{0}^{t} \bigl\Vert \sqrt{\mu} S\bigl( u (s) \bigr) \bigr\Vert _{0}^{2} \,{d}s. \end{aligned} $$

In addition, by (2.8) we have

2.15 $$g〚\rho 〛|u_{3}{|}_{0}^{2}\leq \Lambda^{2}{∥\sqrt{\rho }u∥}_{0}^{2}+\frac{1}{2}{∥\sqrt{\Lambda \mu +\kappa \rho }S(u)∥}_{0}^{2}.$$

Thus, we infer by (2.13)–(2.15) that

2.16 $$ \begin{aligned}[b] &\frac{1}{\Lambda} \Vert \sqrt{ { \rho}} u_{t} \Vert ^{2}_{0}+ \frac{1}{2} \bigl\Vert \sqrt{\mu} S\bigl( u (t)\bigr) \bigr\Vert _{0}^{2} \\ &\quad \leq {\Lambda} \bigl\Vert \sqrt{ {\rho}} u (t) \bigr\Vert ^{2}_{0}+ {\Lambda} \int _{0}^{t} \bigl\Vert \sqrt{\mu} S\bigl( u(s) \bigr) \bigr\Vert _{0}^{2} \,{d}s +\frac{I_{0}+ \Lambda \Vert \sqrt{\mu}S( u_{0}) \Vert _{0}^{2} }{\Lambda}. \end{aligned} $$

Recalling that

$$ \begin{aligned} \Lambda\,\frac{{d}}{{d}t} \Vert \sqrt{ {\rho}} u \Vert ^{2}_{0} = 2\Lambda \int\rho u(t)\cdot u_{t}\,{d} y\leq \Vert \sqrt{\rho} u_{t} \Vert ^{2}_{0} +\Lambda^{2} \bigl\Vert \sqrt{ {\rho}} u(t) \bigr\Vert ^{2}_{0}, \end{aligned} $$

we deduce from (2.16) the differential inequality

$$ \begin{aligned} & \frac{{d}}{{d}t} \Vert \sqrt{ {\rho}} u \Vert ^{2}_{0}+ \frac{1}{2} \bigl\Vert \sqrt {\mu} S \bigl( u(t)\bigr) \bigr\Vert _{0}^{2} \\ &\quad \leq2\Lambda \biggl( \bigl\Vert \sqrt{\rho} u(t) \bigr\Vert ^{2}_{0} +\frac{1}{2} \int _{0}^{t} \bigl\Vert \sqrt{\mu} S\bigl(u(s) \bigr) \bigr\Vert _{0}^{2} \,{d}s \biggr) + \frac{I_{0} + \Lambda \Vert \sqrt{\mu} S(u_{0}) \Vert _{0}^{2} }{\Lambda}. \end{aligned} $$

Applying Gronwall’s inequality [20, Lemma 1.2] to this inequality, we conclude

$$ \begin{aligned} & \bigl\Vert \sqrt{ {\rho}} u (t) \bigr\Vert ^{2}_{0}+\frac{1}{2} \int_{0}^{t} \bigl\Vert \sqrt{\mu} S\bigl( u(s) \bigr) \bigr\Vert ^{2}_{0} \,{d}s \leq \biggl( \Vert \sqrt{ {\rho}}u_{0} \Vert _{0}^{2}+\frac{ I_{0}+ \Lambda \Vert \sqrt {\mu} S( u_{0}) \Vert _{0}^{2} }{2\Lambda^{2}} \biggr) e^{2\Lambda t}, \end{aligned} $$

which, together with (2.16), yields

$$ \begin{aligned} \frac{1}{\Lambda} \bigl\Vert \sqrt{ {\rho}} u_{t} (t) \bigr\Vert ^{2}_{0} + \frac{1}{2} \bigl\Vert \sqrt{\mu} S\bigl( u (t)\bigr) \bigr\Vert _{0}^{2} \leq{}& 2 \biggl(\Lambda \Vert \sqrt{ { \rho}}u_{0} \Vert _{0}^{2}+\frac{ I_{0}+ \Lambda \Vert \sqrt{\mu} S( u_{0}) \Vert _{0}^{2} }{2\Lambda} \biggr) e^{2\Lambda t} \\ &{} +\frac{I_{0}+ \Lambda \Vert \sqrt{\mu} S( u_{0}) \Vert _{0}^{2} }{\Lambda}. \end{aligned} $$

Hence (1.22) follows from the above two estimates and Korn’s inequality. Finally, from (2.8)_1_ we get

$$ \begin{aligned} \bigl\Vert \eta(t) \bigr\Vert _{1} &\lesssim \Vert \eta_{0} \Vert _{1} + \int_{0}^{t} \Vert \eta_{s} \Vert _{1} \,{d}s \lesssim \Vert \eta_{0} \Vert _{1} + \int_{0}^{t} \bigl\Vert u(s) \bigr\Vert _{1}\,{d}s \\ &\lesssim e^{\Lambda t}\bigl( \bigl\Vert (u_{0}, \eta_{0}) \bigr\Vert _{1}+ \sqrt{I_{0}}\bigr) \end{aligned} $$

(i.e., (1.23)), which completes the proof. □

Recalling Definition 1.1, we see that Λ is the largest growth rate of RT instability in the linearized stratified VRT problem from (2.10) and Proposition 2.2. To emphasize the dependence of $\alpha(s)$, Λ, and $\mathcal{G}$ upon *κ*, we denote them by $\alpha(s,\kappa)$, $\Lambda_{\kappa}$, and $\mathcal{G}_{\kappa}$, respectively.

To complete the proof of Theorem 1.1, we further derive relations (1.24) and (1.25) of elasticity coefficient and the largest growth rate. To this end, we need the following auxiliary conclusions.

### Lemma 2.4

*Assume that* $\mu>0$, $\rho>0$, $g>0$, *and* 〚ρ〛>0, *and that the following assertions hold*.

1. *Strict monotonicity*: *if* $\kappa_{1}$ *and* $\kappa_{2}$ *are constants satisfying* $0\leq\kappa_{1}<\kappa_{2}$, *then*
  2.17 $$ \alpha(s,\kappa_{2})< \alpha(s,\kappa_{1}) $$
  *for any given* $s>0$. *Moreover*,
  2.18 $$ \mathcal{G}_{\kappa_{1}}>\mathcal{G}_{\kappa_{2}}, $$
  *where*
  2.19 $$ \mathcal{G}_{\kappa_{i}}:=\sup\bigl\{ s\in\mathbb{R}|\alpha(\tau ,\kappa_{i})>0\textit{ for any }\tau\in(0,s)\bigr\} \quad \textit{and}\quad \alpha ( \mathcal{G}_{\kappa_{i}},\kappa_{i})=0. $$
2. *Continuity*: *for given* $s>0$, $\alpha(s,\kappa)\in C^{0,1}_{\mathrm{loc}}[0,\kappa_{{c}})$ *with respect to the variable* *κ*. (3) *Estimate for* $\mathcal{G}_{\kappa}$: $\mathcal {G}_{\kappa}\leq m$, *where* *κ* *is a constant*.

### Proof

(1) Let $s>0$ be fixed, and let $0\leq\kappa_{1}<\kappa_{2}$. Then by Lemma 2.1 there exists functions $\tilde{u}^{\kappa _{i}}\in H^{\infty}\cap\mathcal{A}$, $i=1,2$, such that

$$\begin{array}{rl} \alpha (s,\kappa_{i}) & =g〚\rho 〛|{\tilde{u}}_{3}^{\kappa_{i}}{|}_{0}^{2}-\frac{s}{2}{∥\sqrt{\mu }S\left({\tilde{u}}^{\kappa_{i}}\right)∥}_{0}^{2}-\frac{1}{2}\kappa {∥\sqrt{\rho }S\left({\tilde{u}}^{\kappa_{i}}\right)∥}_{0}^{2}\\ & :=\hat{E}\left({\tilde{u}}^{\kappa_{i}}\right)-\kappa \Psi \left({\tilde{u}}^{\kappa_{i}}\right), \end{array}$$

where $\hat{E}({\tilde{u}}^{\kappa_{i}}):=g〚\rho 〛{\left|{\tilde{u}}_{3}^{\kappa_{i}}\right|}_{0}^{2}-s{∥\sqrt{\mu }S({\tilde{u}}^{\kappa_{i}})∥}_{0}^{2}/2$, $\Psi(\tilde {u}^{\kappa_{i}}):= \|\sqrt{\rho}S(\tilde{u}^{\kappa_{i}})\|_{0}^{2}/2$, and $\tilde{u}^{\kappa_{i}}_{3}$ denotes the third component of $\tilde {u}^{\kappa_{i}}$. Since $\tilde{u}^{\kappa_{i}}\in\mathcal{A}$, by Korn’s inequality we have

$$ \begin{aligned} 0< \bigl\Vert \tilde{u}^{\kappa_{i}} \bigr\Vert _{{1}}^{2}\lesssim \bigl\Vert S\bigl(\tilde{u}^{\kappa _{i}} \bigr) \bigr\Vert _{0}^{2},\quad i=1,2, \end{aligned} $$

and thus

$$ \begin{aligned} \alpha(s,\kappa_{2})\leq\alpha(s, \kappa_{1})+\frac{(\kappa _{1}-\kappa_{2}) }{2} \bigl\Vert \sqrt{\rho}S\bigl( \tilde{u}^{\kappa_{2}}\bigr) \bigr\Vert _{0}^{2}< \alpha(s, \kappa_{1}). \end{aligned} $$

This yields the desired conclusion (2.17).

Next, we prove (2.18) by contradiction. If $\mathcal{G}_{\kappa _{1}}<\mathcal{G}_{\kappa_{2}}$, then we get from (2.17) and the strict monotonicity of $\alpha(s,\kappa)$ with respect to *s* that

$$ \begin{aligned} 0=\alpha(\mathcal{G}_{\kappa_{2}}, \kappa_{2}) < \alpha(\mathcal{G}_{\kappa_{2}},\kappa_{1}) < \alpha(\mathcal{G}_{\kappa_{1}},\kappa_{1})=0, \end{aligned} $$

which is a paradox. If $\mathcal{G}_{\kappa_{1}}=\mathcal{G}_{\kappa _{2}}$, then exploiting (2.17), we have

$$ \begin{aligned} 0=\alpha(\mathcal{G}_{\kappa_{2}}, \kappa_{2})< \alpha(\mathcal{G}_{\kappa_{2}},\kappa_{1})= \alpha(\mathcal{G}_{\kappa_{1}},\kappa_{1})=0, \end{aligned} $$

which is also a paradox. Thus we immediately get the desired conclusion.

(2) Let $s>0$ be fixed. We choose bounded intervals $[b_{3},b_{4}]\subset (0,\infty)$ and $[b_{5},b_{6}]\subset[0,\kappa_{{c}})$ such that $s\in [b_{3},b_{4}]$. Then, for the given constant $s>0$ and any $\kappa\in [b_{5},b_{6}]$, there is a function $\tilde{w}^{\kappa}\in\mathcal{A}$ satisfying $\alpha(s,\kappa)=\hat{E}(\tilde{w}^{\kappa})-\kappa\Psi (\tilde{w}^{\kappa})$. Thus, in view of the monotonicity of $\alpha (s,\kappa)$, we know that

$$ \begin{aligned} \alpha(b_{4},b_{6})+b_{3} \Phi\bigl(\tilde{w}^{\kappa}\bigr)/2&\leq\alpha(b_{4},\kappa )+b_{3}\Phi\bigl(\tilde{w}^{\kappa}\bigr)/2\leq\tilde{E}\bigl( \tilde{w}^{\kappa}\bigr)-(s/2)\Phi\bigl(\tilde{w}^{\kappa}\bigr) \\ &\leq \alpha(s/2,\kappa)\leq\alpha(b_{3}/2,\kappa)\leq \alpha(b_{3}/2,b_{5}), \end{aligned} $$

which yields

$$\begin{aligned} \Psi\bigl(\tilde{w}^{\kappa}\bigr)&\leq \frac{\max\{\rho_{-},\rho_{+}\}}{ \min\{\mu_{-},\mu_{+}\}}\Phi\bigl(\tilde{w}^{\kappa}\bigr) \\ &\leq2 \bigl(\alpha(b_{3}/2,b_{5})-\alpha(b_{4},b_{6}) \bigr)\frac{\max\{\rho_{-},\rho_{+}\}}{b_{3}\min\{\mu_{-},\mu_{+}\}}:=K' \quad \mbox{for any } \kappa \in[b_{5},b_{6}]. \end{aligned} $$

Thus, for any $\kappa_{1}$, $\kappa_{2}\in[b_{5},b_{6}]$,

$$\alpha(s,\kappa_{1})-\alpha(s,\kappa_{2})\leq\hat{E}\bigl( \tilde {w}^{\kappa_{1}}\bigr)-\kappa_{1}\Psi\bigl( \tilde{w}^{\kappa_{1}}\bigr) - \bigl(\hat{E}\bigl(\tilde{w}^{\kappa_{1}} \bigr)-\kappa_{2}\Psi\bigl(\tilde {w}^{\kappa_{1}}\bigr) \bigr)\leq K' \vert \kappa_{2}-\kappa_{1} \vert . $$

Reversing the role of indices 1 and 2 in the derivation of this inequality, we obtain the same boundedness with the indices switched. Therefore, we deduce that

$$ \begin{aligned} \bigl\vert \alpha(s,\kappa_{1})- \alpha(s,\kappa_{2}) \bigr\vert \leq K' \vert \kappa _{1}-\kappa_{2} \vert , \end{aligned} $$

which yields $\alpha(s,\kappa)\in C^{0,1}_{{\rm loc}}[0,\kappa_{{c}})$.

(3) Recalling the definition of $\kappa_{ c}$, we see that

$$\begin{array}{r} g〚\rho 〛|{\tilde{w}}_{3}{|}_{0}^{2}\leq \frac{\kappa_{c}}{2}{∥\sqrt{\rho }S(\tilde{w})∥}_{0}^{2}\;\text{for any }\tilde{w}\in A. \end{array}$$

Hence, by the first assertion of Lemma 2.1, for any given *κ*, there exists $\tilde{w}^{\kappa}\in\mathcal{A}$ such that

$$\begin{array}{rl} \alpha (s,\kappa ) & =g〚\rho 〛|{\tilde{w}}_{3}^{\kappa }{|}_{0}^{2}-\frac{1}{2}{∥\sqrt{\kappa \rho }S\left({\tilde{w}}^{\kappa }\right)∥}_{0}^{2}-\frac{s}{2}{∥\sqrt{\mu }S\left({\tilde{w}}^{\kappa }\right)∥}_{0}^{2}\\ & \leq \frac{1}{2}\left({∥\sqrt{\rho \kappa_{c}}S\left({\tilde{w}}^{\kappa }\right)∥}_{0}^{2}-{∥\sqrt{\rho \kappa +s\mu }S\left({\tilde{w}}^{\kappa }\right)∥}_{0}^{2}\right). \end{array}$$

Thus, if $\alpha(s,\kappa)\geq0$, then

$$ \begin{aligned} s\leq m \end{aligned} $$

(refer to (1.25) for the definition of *m*). This means that $\mathcal{G}_{\kappa}\leq m$ by the definition of $\mathcal{G}_{\kappa}$ in (2.19). This completes the proof. □

Now we are in position to prove the properties of $\Lambda_{\kappa}$ stated in (1.24) and (1.25) by three steps.

First, we verify the monotonicity of $\Lambda_{\kappa}$ in the variable *κ*. For given two constants $\kappa_{1}$ and $\kappa _{2}$ satisfying $0\leq\kappa_{1}<\kappa_{2}$, there exist two associated curve functions $\alpha(s,\kappa_{1})$ and $\alpha (s,\kappa_{2})$ defined in $(0,\kappa_{{c}})$. By the first assertion in Lemma 2.4,

$$ \begin{aligned} \alpha(s,\kappa_{1})>\alpha(s, \kappa_{2}). \end{aligned} $$

On the one hand, the fixed point $\Lambda_{\kappa_{i}}$ satisfying $\Lambda_{\kappa_{i}}=\sqrt{\alpha(\Lambda_{\kappa_{i}})}$ can be obtained as the intersection point of the two curves $y=s$ and $y=\alpha(s,\kappa_{i})$ for $i=1$ and 2. Thus we can immediately observe the monotonicity:

2.20 $$ \begin{aligned} \Lambda_{\kappa_{1}}> \Lambda_{\kappa_{2}}\quad \mbox{for }0\leq\kappa _{1}< \kappa_{2}. \end{aligned} $$

Second, we show the continuity. To this end, we choose a constant $\kappa_{0}>0$ and an associated function $\alpha(s,\kappa_{0})$. Noting that $\alpha(\Lambda_{\kappa_{0}},\kappa_{0})=\Lambda ^{2}_{\kappa_{0}}>0$ and $\alpha(s,\kappa_{0})\in C^{0,1}_{{\rm loc}}[0,\kappa_{{c}})$ are strictly decreasing with respect to *κ*, we have that, for any given $\varepsilon>0$, there exists a constant $\delta>0$ such that

$$ \begin{aligned} \alpha(\Lambda_{\kappa_{0}},\kappa_{0}+ \delta)>0,\quad0< \sqrt {\alpha(\Lambda_{\kappa_{0}},\kappa_{0})}- \sqrt{\alpha(\Lambda _{\kappa_{0}},\kappa_{0}+\delta)}< \varepsilon \end{aligned} $$

and

$$ \begin{aligned} 0< \sqrt{\alpha(\Lambda_{\kappa_{0}},\kappa _{0}-\delta)}-\sqrt{\alpha(\Lambda_{\kappa_{0}},\kappa _{0})}< \varepsilon. \end{aligned} $$

In particular, we have

$$ \begin{aligned} \Lambda_{\kappa_{0}}-\varepsilon< \sqrt{\alpha( \Lambda_{\kappa _{0}},\kappa_{0}+\delta)}\quad \mbox{and}\quad \sqrt{\alpha( \Lambda_{\kappa _{0}},\kappa_{0}-\delta)}< \Lambda_{\kappa_{0}}+ \varepsilon. \end{aligned} $$

By the monotonicity of $\Lambda_{\kappa}$ with regard to *κ* we get

$$ \begin{aligned} \Lambda_{\kappa_{0}-\delta}>\Lambda_{\kappa_{0}}> \Lambda_{\kappa _{0}+\delta}. \end{aligned} $$

Thus, using the monotonicity of $\alpha(s,\kappa)$ with regard to *s*, we obtain

$$ \begin{aligned} \sqrt{\alpha(\Lambda_{\kappa_{0}}, \kappa_{0}+\delta)}< \sqrt {\alpha(\Lambda_{\kappa_{0}+\delta}, \kappa_{0}+\delta)}=\Lambda _{\kappa_{0}+\delta} \end{aligned} $$

and

$$ \begin{aligned} \sqrt{\alpha(\Lambda_{\kappa_{0}}, \kappa_{0}-\delta)} >\sqrt{\alpha(\Lambda_{\kappa_{0}-\delta}, \kappa_{0}-\delta)}=\Lambda_{\kappa_{0}-\delta}. \end{aligned} $$

Chaining the five inequalities above, we immediately get

$$ \begin{aligned} \Lambda_{\kappa_{0}}-\varepsilon< \Lambda_{\kappa_{0}+\delta }< \Lambda_{\kappa_{0}-\delta}< \Lambda_{\kappa_{0}}+ \varepsilon. \end{aligned} $$

Then, for any $\kappa\in(\kappa_{0}-\delta,\kappa_{0}+\delta)$, we arrive at

$$ \begin{aligned} \Lambda_{\kappa_{0}}-\varepsilon< \Lambda_{\kappa}< \Lambda_{\kappa _{0}}+\varepsilon. \end{aligned} $$

Similarly, for $\kappa_{0}=0$, we also can prove that, for any $\varepsilon>0$, there exists $\delta>0$ such that, for any $\kappa \in[\kappa_{0},\kappa_{0}+\delta)\subset[0,\kappa_{{c}})$,

$$ \begin{aligned} \Lambda_{\kappa_{0}}< \Lambda_{\kappa}< \Lambda_{\kappa _{0}}+\varepsilon. \end{aligned} $$

Consequently, we get the conclusion

2.21 $$ \begin{aligned} \Lambda_{\kappa} \mbox{ is a continuous function of } \kappa\in[0,\kappa_{{c}}). \end{aligned} $$

Third, noting that $\Lambda_{\kappa}\in(0,\mathcal{G}_{\kappa})$, we immediately get

2.22 $$ \begin{aligned} \Lambda_{\kappa}\leq m \end{aligned} $$

by the third conclusion in Lemma 2.4. Consequently, we complete the proof of Theorem 1.1 from (2.20)–(2.22) and Propositions 2.1–2.2.

## Extension to MHD fluids

Recently, Wang [24] established the nonlinear stability and linear instability of the stratified magnetic Rayleigh–Taylor (MRT) problem. His result shows that magnetic fields can also inhibit the development of RT instability. In this section, we extend the obtained result in linearized stratified VRT problem to the linearized stratified MRT problem. More precisely, we establish qualitative relations between magnetic fields and the largest growth rates in the linear RT instability of stratified MRT problem. Next, we introduce the stratified MRT problem investigated by Wang. In what follows, we continue to use the mathematical notations of Sect. 1, unless specified otherwise.

The three-dimensional motion equations of incompressible MHD fluids without resistivity under a uniform gravitational field (along the negative $x_{3}$-direction) can be described as follows [10, 12, 15]:

3.1 $$ \textstyle\begin{cases} \rho_{t}+\operatorname{div}(\rho{ v})=0 ,\\ \rho v_{t}+\rho v\cdot\nabla v+\operatorname{div}\mathcal{S}(p^{g},v,M)=-\rho g e_{3},\\ M_{t}=M\cdot\nabla v-v\cdot\nabla M , \\ \operatorname{div}v=\operatorname{div}M=0, \end{cases} $$

where *M* denotes the magnetic field, and stress tension $\mathcal {S}(p^{g},v,M)$ is defined as follows:

$$\mathcal{S}\bigl(p^{g},v,M\bigr) :=-\mu{S}(v)+{p}^{g}I + \lambda\bigl( \vert M \vert ^{2}I/2-M\otimes M\bigr). $$

Here *λ* is the permeability of vacuum divided by 4*π*.

Referring to (1.1), we can easily use these motion equations to establish the mathematical model of the motion of the stratified incompressible MHD fluids without resistivity under a uniform gravitational field [16]:

3.2 $$\left\{ \begin{array}{cc} \rho_{\pm }(\partial_{t}v_{\pm }+v_{\pm }\cdot \nabla v_{\pm })+\mathrm{div}S_{\pm }(p_{\pm }^{g},v_{\pm },M_{\pm })=0 & \text{in }\;\Omega_{\pm }(t),\\ \partial_{t}M_{\pm }+v_{\pm }\cdot \nabla M_{\pm }=M_{\pm }\cdot \nabla v_{\pm } & \text{in }\Omega_{\pm }(t),\\ \mathrm{div}v_{\pm }=\mathrm{div}M_{\pm }=0 & \text{in }\Omega_{\pm }(t),\\ d_{t}+v_{1}\partial_{1}d+v_{2}\partial_{2}d=v_{3} & \text{on }T,\\ 〚v_{\pm }〛=0,\;〚S(p_{\pm }^{g},v_{\pm },M_{\pm })〛\nu =gd〚\rho 〛\nu & \text{on }\Sigma (t),\\ v_{\pm }{|}_{t=0}=v_{\pm }^{0},\;M_{\pm }{|}_{t=0}=M_{\pm }^{0} & \text{in }\Omega_{\pm }(0),\\ d{|}_{t=0}=d_{0} & \text{on }T. \end{array}\right.$$

To investigate the RT instability in (3.2), we assume that 〚ρ〛>0. Then we call this model the stratified MRT problem. Since the upper fluid is heavier than the lower fluid, the stratified MRT problem may be unstable due to the RT instability. The initial-boundary problem (3.2) admits an equilibrium-state solution with $v=0$, $p=\mbox{const}$, $M=\bar{M}$, and $d=\bar{d}$, where *M̄* is a uniform magnetic field. Without loss of generality, we assume that $\bar{d}=0$.

Let *ζ* and *η* be defined by (1.9) and $\eta :=\zeta-y$. Similarly to (1.10), using the additional definition

$$B:=\bigl(M_{+}(x,t)\chi_{+}+M_{-}(x,t)\chi_{-}\bigr)|_{x=\zeta(y,t)}, $$

the stratified MRT problem (3.2) in Lagrangian coordinates reads as follows:

3.3 $$\left\{ \begin{array}{cc} \eta_{t}=u & \text{in }\Omega ,\\ \rho u_{t}+{\mathrm{div}}_{A}S_{A}(q,u)=\lambda B\cdot \nabla_{A}B & \text{in }\Omega ,\\ B_{t}=B\cdot \nabla_{A}u & \text{in }\Omega ,\\ {\mathrm{div}}_{A}u={\mathrm{div}}_{A}B=0 & \text{in }\Omega ,\\ 〚u〛=0,\;〚S_{A}(q,u)〛\vec{n}=\lambda 〚B\cdot \vec{n}B〛+g〚\rho 〛\eta_{3}\vec{n} & \text{on }\Sigma ,\\ u=0 & \text{on }\Sigma_{-}^{+},\\ \left(\eta ,u,B){|}_{t=0}=(\eta_{0},u_{0},B_{0}\right) & \text{in }\Omega , \end{array}\right.$$

where we have defined

3.4 $$\begin{aligned} \mathcal{S}_{\mathcal{A}}(q,u):=qI-\mu\mathcal{S}_{\mathcal{A}}(u). \end{aligned}$$

The initial-boundary problem (3.3) admits an equilibrium-state solution with $\zeta=y$, $u=0$, $q=\mathrm{const}$, and $B=\bar{M}$. To investigate the stability and instability of the initial-boundary problem (3.3) around the equilibrium-state solution, as in Wang [24], we further assume that

3.5 $$ \zeta_{0}=y,\quad \mbox{ i.e., }\eta_{0}=0; $$

then we have $\det(\nabla\zeta_{\pm})=1$.

Next, we recall some conservation relations involving the magnetic field. Applying $\mathcal{A}^{\mathrm{T}}$ to the magnetic induction equation (3.3)_3_ and using (1.15), we obtain

3.6 $$ \begin{aligned} \mathcal{A}_{ji} \partial_{t}B_{j} =\mathcal{A}_{ji}B_{k} \mathcal{A}_{kl} \partial_{l}u_{j}= \mathcal{A}_{ji}B_{k} \mathcal{A}_{kl} \partial_{t}(\partial_{l} \zeta_{j})=- \partial_{t}\mathcal{A}_{ji}B_{k} \mathcal{A}_{kl}\partial_{l}\zeta_{j}=-B_{j} \partial_{t}\mathcal{A}_{ji}. \end{aligned} $$

This implies that

3.7 $$ \begin{aligned} \partial_{t}\bigl( \mathcal{A}^{\mathrm{T}}B\bigr)=0. \end{aligned} $$

It then follows from (1.16), $\operatorname{div}_{\mathcal{A}}B=0$, and (3.7) that

3.8 $$ \begin{aligned} \partial_{t}( \mathrm{div}_{\mathcal{A}}B)= \partial_{t}\mathrm {div}\bigl( \mathcal{A}^{\mathrm{T}}B\bigr)=0 \end{aligned} $$

and

3.9 $$ \begin{aligned} \partial_{t} \bigl(B_{\pm}\cdot(\mathcal{A}_{\pm}e_{3})\bigr) = \partial_{t}\bigl(e_{3}^{{T}}\mathcal{A}^{\mathrm{T}}_{\pm} {B}_{\pm}e_{3}\bigr)=0 \quad\mbox{on }\Sigma. \end{aligned} $$

Hence by (3.5) we can derive from (3.7)–(3.9) that

3.10 $$ \begin{aligned} \mathcal{A}^{\mathrm{T}}B=\bar{M},\qquad \mathrm{div}_{\mathcal {A}}B=0\quad \mbox{in } \Omega\quad \mbox{and}\quad B_{\pm}\cdot( \mathcal{A}_{\pm}e_{3})=\bar {M}_{3}\quad \mbox{on } \Sigma. \end{aligned} $$

The conservation analysis above reveals that the magnetic field *B* should have certain relations with the flow map *ζ*. In turn, this motivates us to eliminate the magnetic field *B* in the initial-boundary values problem (3.3). Indeed, since $B=\bar{M}\cdot\nabla\zeta$ by the first identity in (3.10), we may rewrite the generalized Lorentz force term:

3.11 $$ \begin{aligned} B\cdot\nabla_{\mathcal{A}}B=B_{j} \mathcal{A}_{jk} \partial_{k}B=\bar{M}_{k} \partial_{k}(\bar{M}_{i} \partial_{i}\zeta)=( \bar{M}\cdot\nabla)^{2}\zeta. \end{aligned} $$

Consequently, the initial-boundary values problem (3.3) can be reformulated as follows:

3.12 $$\left\{ \begin{array}{cc} \eta_{t}=u & \text{in }\Omega ,\\ \rho u_{t}+{\mathrm{div}}_{A}S_{A}(q,u)=\lambda {\left(\bar{M}\cdot \nabla \right)}^{2}\eta & \text{in }\Omega ,\\ {\mathrm{div}}_{A}u=0 & \text{in }\Omega ,\\ 〚u〛=0,\;〚S_{A}(q,u)〛\vec{n}=\lambda 〚{\bar{M}}_{3}(\bar{M}\cdot \nabla )\eta 〛+g〚\rho 〛\eta_{3}\vec{n} & \text{on }\Sigma ,\\ u=0 & \text{on }\Sigma_{-}^{+},\\ \left(\eta ,u){|}_{t=0}=(\eta_{0},u_{0}\right) & \text{in }\Omega . \end{array}\right.$$

We call this problem the transformed stratified MRT problem. The corresponding linearized problem of the transformed stratified MRT problem reads as follows:

3.13 $$\left\{ \begin{array}{cc} \eta_{t}=u & \text{in }\Omega ,\\ \rho u_{t}-\mu \Delta u+\nabla q-\lambda {\left(\bar{M}\cdot \nabla \right)}^{2}\eta =0 & \text{in }\Omega ,\\ \mathrm{div}u=0 & \text{in }\Omega ,\\ 〚u〛=0,\;〚qI-\mu S(u)〛e_{3}-\lambda 〚{\bar{M}}_{3}(\bar{M}\cdot \nabla )\eta 〛=g〚\rho 〛\eta_{3}e_{3} & \text{on }\Sigma ,\\ u=0 & \text{on }\Sigma_{-}^{+}. \end{array}\right.$$

Let $\Omega_{1}:=\mathbb{R}^{2}\times(-l,m)$ and

$$M_{c}:=\sqrt{\frac{〚\rho 〛g}{\lambda (\frac{1}{l}+\frac{1}{m})}}.$$

Wang [24] has proved that the linearized stratified MRT problem defined on $\Omega_{1}$ is stable for $\bar{M}_{3}>\mathcal {M}_{{c}} $ and unstable for $\bar{M}_{3}<\mathcal{M}_{{c}} $. Moreover, he also verified that the nonlinear stratified MRT problem defined on $\Omega_{1}$ is also stable for $\bar{M}_{3}>\mathcal {M}_{{c}} $. The nonlinear stability result shows that the magnetic fields can inhibit the development of RT instability. Of course, Wang’s results also hold for the domain Ω.

### Main result

Before introducing our result, let us recall the derivation of the instability criterion. We consider the following growing mode solutions to (3.13):

3.14 $$ \begin{aligned} \eta(t,x)=\tilde{\eta}(x)e^{\Lambda t}, \qquad u(t,x)=\tilde {u}(x)e^{\Lambda t},\qquad q(t,x)=\tilde{q}(x)e^{\Lambda t} \end{aligned} $$

for some $\Lambda>0$. Substituting the above ansatz into (3.13), we get an eigenvalue problem

3.15 $$\left\{ \begin{array}{cc} \Lambda \tilde{\eta }=\tilde{u} & \text{in }\Omega ,\\ \rho \Lambda \tilde{u}-\mu \Delta \tilde{u}+\nabla \tilde{q}-\lambda {\left(\bar{M}\cdot \nabla \right)}^{2}\tilde{\eta }=0 & \text{in }\Omega ,\\ \mathrm{div}\tilde{u}=0 & \text{in }\Omega ,\\ 〚\tilde{u}〛=0,\;〚\tilde{q}I-\mu S(\tilde{u})〛e_{3}-\lambda 〚{\bar{M}}_{3}(\bar{M}\cdot \nabla )\tilde{\eta }〛=g〚\rho 〛{\tilde{\eta }}_{3}e_{3} & \text{on }\Sigma ,\\ \tilde{u}=0 & \text{on }\Sigma_{-}^{+}. \end{array}\right.$$

By using (3.15)_1_ we can eliminate *η̃* and arrive at the following boundary-value problem for blue$(\tilde{u},\tilde{q})$:

3.16 $$\left\{ \begin{array}{cc} \Lambda^{2}\rho \tilde{u}-\Lambda \mu \Delta \tilde{u}+\Lambda \nabla \tilde{q}-\lambda {\left(\bar{M}\cdot \nabla \right)}^{2}\tilde{u}=0 & \text{in }\Omega ,\\ \mathrm{div}\tilde{u}=0 & \text{in }\Omega ,\\ 〚\tilde{u}〛=0,\;〚\tilde{q}I-\Lambda \mu S(\tilde{u})〛e_{3}-\lambda 〚{\bar{M}}_{3}(\bar{M}\cdot \nabla )\tilde{u}〛=〚\rho 〛g{\tilde{u}}_{3}e_{3} & \text{on }\Sigma ,\\ \tilde{u}=0 & \text{on }\Sigma_{-}^{+}. \end{array}\right.$$

This boundary-value problem enjoys the following energy structure:

3.17 $$ \begin{aligned} \Lambda^{2}J(\tilde{u})=E( \tilde{u},s):=E_{0}(\tilde{u})-s \Phi (\tilde{u}), \end{aligned} $$

where

3.18 $$\begin{array}{r} E_{0}(\tilde{u}):=g〚\rho 〛|{\tilde{u}}_{3}{|}_{0}^{2}-\lambda {∥\bar{M}\cdot \nabla \tilde{u}∥}_{0}^{2}. \end{array}$$

Thus, similarly to the instability criterion of the linearized stratified VRT problem, we easily get that if

3.19 $$\begin{array}{r} 0<|{\bar{M}}_{3}|<\bar{M_{c}}:=\sqrt{\underset{\tilde{u}\in H_{\sigma }^{1}}{\sup}\frac{g〚\rho 〛|{\tilde{u}}_{3}{|}_{0}^{2}}{\lambda {∥{\bar{M}}^{*}\cdot \nabla \tilde{u}∥}_{0}^{2}}}, \end{array}$$

then the linearized stratified MRT problem (3.13) is unstable, where $\bar{M}^{*}:=\bar{M}/\bar{M}_{3}$. In fact, recently Jiang et al. have proved that under condition (3.19) or $\bar{M}_{3}=0$, the linearized stratified MRT problem (3.13) is unstable from the well-known minimum potential energy principle. Moreover, they also prove that

3.20 $$ \begin{aligned} \overline{\mathcal{M}_{{c}}}= \mathcal{M}_{{c}}; \end{aligned} $$

refer to [11, Lemma 4.5].

Finally, we introduce the largest growth rate of RT instability in the linearized stratified MRT problem.

#### Definition 3.1

We call $\Lambda>0$ the largest growth rate of RT instability in the linearized stratified MRT problem if it satisfies the following two conditions:

1. For any classical solution $(u,\eta)$ of the linearized stratified MRT problem with an associated pressure *q*, we have, for any $t\geq0$,
  3.21 $$\begin{aligned} & \bigl\Vert u(t) \bigr\Vert _{1}^{2}+ \Vert u_{t} \Vert ^{2}_{0 }+ \int _{0}^{t} \bigl\Vert u(s) \bigr\Vert ^{2}_{1}\,{d}s\lesssim e^{2\Lambda t}\bigl( \Vert u_{0} \Vert _{1}^{2} +I_{0}\bigr), \end{aligned}$$
  3.22 $$\begin{aligned} & \bigl\Vert \eta(t) \bigr\Vert _{1} \lesssim e^{\Lambda t}\bigl( \bigl\Vert (u_{0}, \eta_{0}) \bigr\Vert _{1}+\sqrt{I_{0}}\bigr), \end{aligned}$$
  where $I_{0}:=\|\sqrt{\rho}u_{t}(0)\|^{2}_{0} -{E}_{0}(u_{0})$.
2. There exists a solution $(u,\eta,q)$ of the linearized stratified MRT problem in the form
  $$(u,\eta,q):=e^{\Lambda t}(\tilde{u},\tilde{\eta},\tilde{q}), $$
  where $(\tilde{u},\tilde{\eta},\tilde{q})\in H^{2}\times H^{2}\times H^{1}$.

In this paper, for $\bar{M}:=(0,0,\mathcal{M})$, we further prove that the largest growth rate Λ in the linearized stratified MRT problem decreases from a positive constant to 0 as $|\bar{M}_{3}|$ increases from 0 to $\mathcal{M}_{{c}}$. More precisely, we have the following conclusion.

#### Theorem 3.1

*Let* *g*, *μ*, *ρ*, *and* $\bar{M}:=(0,0,\mathcal{M})$ *be given*. *We further assume that* 〚ρ〛>0 *and* $|\mathcal {M}|<\mathcal{M}_{{c}}$. *Then there exists a largest growth rate* $\Lambda>0$ (*see Definition* 3.1) *such that there exists an unstable solution to the linearized stratified MRT problem* (3.12) *of the form*

$$(u,\eta,q):=e^{\Lambda t}(\tilde{u},\tilde{u}/\Lambda,\tilde{q}), $$

*where* $(\tilde{u},\tilde{q})\in(H^{1}_{\sigma}\cap H^{\infty})\times H^{\infty}$ *solves the boundary value problem* (3.16). *Moreover*, *for given* *g*, *μ*, *and* *ρ*, *we can regard* $\Lambda _{\mathcal{M}}:=\Lambda$ *as a function of* $\mathcal {M}\in(-\mathcal{M}_{{c}},\mathcal{M}_{{c}})$, *which enjoys the following properties*:

3.23 $$\begin{aligned} &\Lambda_{\mathcal{M}}=\Lambda_{-\mathcal{M}}, \end{aligned}$$

3.24 $$\begin{aligned} & \Lambda_{\mathcal{M}}\textit{ strictly decreases on }[0, \mathcal{M}_{{c}}), \quad\Lambda_{\mathcal{M}}\in C^{0}(- \mathcal{M}_{{c}},\mathcal{M}_{{c}}), \end{aligned}$$

3.25 $$\begin{aligned} & \Lambda_{\mathcal{M}}\leq\lambda\bigl(\mathcal {M}_{{c}}^{2}-\mathcal{M}^{2}\bigr)/\min\{\mu_{-}, \mu_{+}\}. \end{aligned}$$

We can follow the argument of Theorem 1.1 to derive Theorem 3.1. The detailed proof of Theorem 3.1 is provided in the next subsection.

### Proof of Theorem 3.1

Next, we follow the argument of Theorem 1.1 to prove Theorem 3.1. To begin with, we modify (3.16) as follows:

3.26 $$\left\{ \begin{array}{cc} \rho \beta (s)\tilde{w}-s\mu \Delta \tilde{w}+s\nabla \tilde{q}-\lambda M^{2}\partial_{3}^{2}\tilde{w}=0 & \text{in }\Omega ,\\ \mathrm{div}\tilde{w}=0 & \text{in }\Omega ,\\ 〚\tilde{w}〛=0,\;〚\tilde{q}I-s\mu S(\tilde{w})〛e_{3}-\lambda M^{2}〚\partial_{3}\tilde{w}〛=〚\rho 〛g{\tilde{w}}_{3}e_{3} & \text{on }\Sigma ,\\ \tilde{w}=0 & \text{on }\Sigma_{-}^{+}. \end{array}\right.$$

Then we can look for $(\beta(s), \tilde{w} )$ from the variational problem

3.27 $$ \beta(s)=\sup_{{\tilde{u}}\in\mathcal{A}} {E}(\tilde{u},s) \in \mathbb{R}. $$

More precisely, we have the following conclusions.

#### Lemma 3.1

*Under the assumptions of Theorem* 3.1, *for any but fixed* $s>0$, *the following assertions are valid*.

1. *In the variational problem* (3.27), ${E}(\tilde{u})$ *achieves its supremum on* $\mathcal{A}$.
2. *Let* $\tilde{u}_{0}$ *be a maximizer*, *and let* $\beta:=\sqrt{\sup_{\tilde{u}\in\mathcal{A}} {E}(\tilde{u})}$. *Then there exists a pressure function* $\tilde{q}_{0}$ *associated with* $\tilde{u}_{0}$ *such that the triple* $(\tilde{u}_{0},\tilde{q}_{0},\beta)$ *satisfies the boundary problem* (3.26). *Moreover*, $(\tilde{u}_{0},\tilde {q}_{0})\in(H^{1}_{\sigma}\cap H^{\infty})\cap H^{\infty}$.

#### Proof

(1) Following the argument of the first assertion of Lemma 2.1, we can easily get the first conclusion.

(2) To prove the second assertion, we notice that since ${E}(\tilde {u})$ and $J(\tilde{u})$ are homogeneous of degree 2, (3.27) is equivalent to

3.28 $$ \beta=\sup_{w\in H_{\sigma}^{1}}\frac{ {E}(w)}{J(w)}. $$

For any given $\tau\in\mathbb{R}$ and ${w}\in H_{\sigma}^{1}$, we take $\tilde{ {w}}:=\tilde{{u}}_{0}+\tau{w}$. Then (3.28) implies

$$ {E}(\tilde{ {w}})-\beta J(\tilde{ {w}})\leq0. $$

If we set $I(\tau)= {E}(\tilde{ {w}})-\beta J(\tilde{ {w}})$, then we see that $I(\tau)\in C^{1}(\mathbb{R})$, $I(\tau)\leq0$ for all $\tau\in\mathbb{R}$, and $I(0)=0$. This implies $I'(0)=0$. Hence a direct computation leads to

3.29 $$\begin{array}{rl} & \beta \int \rho {\tilde{u}}_{0}\cdot w\;dy+\lambda M^{2}\int \partial_{3}{\tilde{u}}_{0}\cdot \partial_{3}w\;dy+\frac{s}{2}\int \mu S({\tilde{u}}_{0}):S(w)\;dy\\ & \;=g〚\rho 〛\int_{\Sigma }{\tilde{u}}_{03}w_{3}\;dy_{h}. \end{array}$$

Noting that

$$\int\nabla\tilde{u}_{0}^{{T}}:\nabla \tilde{u}_{0}\,{d}y=0 $$

due to $\operatorname{div}\tilde{u}_{0}=0$, (3.29) can be rewritten as follows:

$$\begin{array}{rl} & \beta \int \rho {\tilde{u}}_{0}\cdot w\;dy+\int \left(s\mu +\lambda M^{2}\right)S({\tilde{u}}_{0}):\nabla w\;dy\\ & \;=\lambda M^{2}\int (\partial_{1}{\tilde{u}}_{0}\cdot \partial_{1}w+\partial_{2}{\tilde{u}}_{0}\cdot \partial_{2}w)\;dy+g〚\rho 〛\int_{\Sigma }{\tilde{u}}_{03}w_{3}\;dy_{h}, \end{array}$$

which implies that $\tilde{u}_{0}\in H^{1}$ is a weak solution to the boundary problem (3.26). Next, we further improve the regularity of *ũ*.

Noting that

$$\Vert v \Vert _{1}^{2}\lesssim \lambda \Vert \mathcal{M} \partial_{3}v \Vert ^{2}_{0} +s \Phi(v)\quad \mbox{for any }v\in H_{0}^{1}, $$

we deduce from the weak form of (3.26) that $\partial _{{h}}\tilde{u}_{0}\in H^{1}_{0}$ by the standard difference quotient method (refer to the derivation of (3.32) in [25]), and thus we further get that $\tilde{u}_{0}\in H^{3/2}(\Sigma)$ by the trace theorem.

Now we consider the following stratified steady Stokes problem:

3.30 $$\left\{ \begin{array}{cc} -(s\mu +\lambda M^{2})\Delta \tilde{u}+s\nabla \tilde{q}=H & \text{in }\Omega ,\\ \mathrm{div}\tilde{u}=0 & \text{in }\Omega ,\\ 〚\tilde{u}〛=0,\;〚s\tilde{q}I-(s\mu +\lambda M^{2})S(\tilde{u})〛e_{3}=F & \text{on }\Sigma ,\\ \tilde{u}=0,\;\tilde{\eta }=0 & \text{on }\Sigma_{-}^{+}. \end{array}\right.$$

It follows from the classical regularity of stratified steady Stokes problem in [24, Lemma A.8] that, if $\mathcal{H}\in H^{m}$ and $\mathcal{F}\in H^{m+1/2}$ for $m\geq0$, then there exists a unique solution $(\tilde{u}, q)\in H^{m+2} \times H^{m+1}$ to (3.30) such that

$$\begin{array}{rl} {∥\tilde{u}∥}_{m+2}+{∥\nabla \tilde{q}∥}_{m}+|〚\tilde{q}〛{|}_{m+1/2} & ≲{∥H∥}_{m}+|F{|}_{m+1/2}. \end{array}$$

Let $\mathcal{H}=-\beta(s)\rho\tilde{u}_{0}-\lambda\mathcal {M}^{2}(\partial_{1}^{2}+\partial_{2}^{2})\tilde{u}_{0}$ and $F=g〚\rho 〛{\tilde{u}}_{03}e_{3}\in H^{1/2}$. Since $\tilde{u}_{0}\in H^{3/2}(\Sigma)$ and $\partial_{{h}}\tilde {u}_{0}\in H^{1}_{0}$, we have $\mathcal{H} \in L^{2}$ and $\mathcal{F}\in H^{1/2}$. Then, by the above regularity result, there exists a solution $(\tilde {u},q)\in H^{2}\times H^{1}$ to (3.30). Since $\tilde{u}_{0}$ is a weak solution to (3.30), we can easily check that $\tilde{u}_{0}=\tilde{u}\in H^{2}$.

Consequently, following the above argument and a standard bootstrap method of improving the regularity, we can easily see that $(\tilde {u}_{0},\tilde{q}_{0})\in(H^{1}_{\sigma}\cap H^{\infty})\times H^{\infty}$. This completes the proof. □

To prove that there is a fixed point Λ such that $\Lambda =\sqrt{\beta(\Lambda)}>0$, we further give some properties of $\beta(s)$ with respect to $s>0$.

#### Lemma 3.2

*Under the assumptions of Theorem* 3.1, *the function* $\beta(s)$ *defined on* $(0,\infty)$ *enjoys the following properties*:

1. $\beta(s)\in C^{0,1}_{\mathrm{loc}}(0,\infty)$ *is strictly decreasing in the variable* *s*.
2. *There are constants* $c_{1}$, $c_{2}>0$, *which depend on* *g*, *ρ*, $\mathcal{M}$, *and* *μ*, *such that*
  3.31 $$ \beta(s)\geq c_{1}-c_{2} s. $$

#### Proof

(1) Following the argument of the first assertion of Lemma 2.2, we can easily get the first conclusion.

(2) If $\mathcal{M} \neq0$, by (3.20) we can deduce from the instability condition $|\mathcal{M}|<\mathcal{M}_{{c}}$ that

$$|M|<\sqrt{\underset{\tilde{u}\in H_{\sigma }^{1}}{\sup}\frac{g〚\rho 〛|{\tilde{u}}_{3}{|}_{0}^{2}}{\lambda {∥\partial_{3}\tilde{u}∥}_{0}^{2}}}.$$

Then we have that

3.32 $$\begin{array}{r} \text{there there exists }\tilde{u}\in H_{\sigma }^{1}\text{ such that }g〚\rho 〛|{\tilde{u}}_{3}{|}_{0}^{2}-\lambda {∥M\partial_{3}\tilde{u}∥}_{0}^{2}>0. \end{array}$$

If $\mathcal{M}=0$, then obviously (3.32) also holds. Consequently, we have

$$ \begin{aligned} \beta(s)&=\sup_{w\in\mathcal{A}} {E}(w,s) = \sup_{w\in H^{1}_{\sigma}}\frac{ {E}(w,s)}{J(w)} \\ &\geq\frac{E_{0}(\tilde{u})}{\int\rho \vert \tilde{u} \vert ^{2}\,{d}y}-\frac{ s \Vert \sqrt{\mu}S(\tilde{u}) \Vert _{0}^{2}/2}{\int\rho \vert \tilde{u} \vert ^{2}\,{d}y}:=c_{1}-sc_{2} \end{aligned} $$

for two positive constants $c_{1}:=c_{1}(g,\rho,\bar{M})$ and $c_{2}:=c_{2}(\mu,\rho)$. This completes the proof of Lemma 3.2. □

Next, we prove that there exists a pair of functions $(\tilde {u},\tilde{q})$ satisfying (3.16) with growth rate Λ by a fixed-point argument.

Let

3.33 $$ \mathcal{G}:=\sup\bigl\{ s|\beta(\tau)>0 \mbox{ for any } \tau \in(0,s)\bigr\} . $$

By Lemma 3.2, $\mathcal{G}>0$; moreover, $\beta(s)>0$ for any $s<\mathcal{G}$. Since $\beta(s)=\sup_{\tilde{u}\in\mathcal{A}} {E}(\tilde{u},s)<\infty $, using the monotonicity of $\beta(s)$, we see that

3.34 $$ \lim_{s\rightarrow0}\beta(s)\quad \mbox{exists, and the limit is a positive constant}. $$

By (2.1) and Korn’s inequality there are two constants $c_{3}$ and $c_{4}$, depending on the domain Ω and other known physical parameters, such that

$$g〚\rho 〛|{\tilde{u}}_{3}{|}_{0}^{2}\leq c_{3}{∥\tilde{u}∥}_{1}^{2}\;\text{for any }\tilde{u}\in A$$

and

$$ \Vert \tilde{u} \Vert _{1}^{2}\leq c_{4} \bigl\Vert S(\tilde{u}) \bigr\Vert _{0}^{2}/2 \quad\mbox{for any }\tilde{u}\in H^{1}_{0}. $$

Thus, if $s>c_{3}c_{4}/\min\{\mu_{-},\mu_{+}\}$, then

$$\begin{array}{rl} & g〚\rho 〛|{\tilde{u}}_{3}{|}_{0}^{2}-\left(\lambda {∥\bar{M}\cdot \nabla \tilde{u}∥}_{0}^{2}+\frac{1}{2}{∥\sqrt{s\mu }S(\tilde{u})∥}_{0}^{2}\right)\\ & \;\leq g〚\rho 〛|{\tilde{u}}_{3}{|}_{0}^{2}-\frac{1}{2}{∥\sqrt{s\mu }S(\tilde{u})∥}_{0}^{2}\\ & \;\leq \left(c_{3}-\frac{s\min\{\mu_{-},\mu_{+}\}}{c_{4}}\right){∥\tilde{u}∥}_{1}^{2}<0\;\text{for any }\tilde{u}\in A, \end{array}$$

which implies that

$$ \beta(s)\leq0\quad\mbox{for any } s>c_{3}c_{4}/\min\{ \mu_{-},\mu_{+}\}. $$

Hence $\mathcal{G}<\infty$. Moreover,

3.35 $$ \lim_{s\rightarrow\mathcal{G}}\beta(s)=0. $$

Similarly to Lemma 2.3, using (3.33)–(3.35) and the continuity of $\beta(s)$ on $(0, \mathcal{G})$, we can deduce that there exists a unique $\Lambda\in(0,\mathcal{G})$ such that

3.36 $$ \Lambda=\sqrt{\beta(\Lambda)}= \sqrt{\sup_{\tilde{w}\in\mathcal{A}} {E}(\tilde{w},\Lambda)}>0. $$

By Lemma 3.1 there is a solution $(\tilde{u},\tilde {q})\in(H^{1}_{\sigma}\cap H^{\infty})\times H^{\infty}$ to problem (3.16) with Λ constructed in (3.36). Moreover, $\Lambda^{2}= {E}(\tilde{u},\Lambda)$, $\tilde{u}_{3}\neq 0$ in Ω, and $\tilde{u}_{3}\neq0$ on Σ by (3.36). Since $\operatorname{div}\tilde{u}=0$ and $\tilde{u}|_{\Sigma ^{+}_{-}}=0$, we immediately get $\tilde{u}^{2}_{1}+\tilde {u}^{2}_{2}\neq0$. In addition, similarly to Proposition 2.1, we can also verify that $\Lambda>0$ is the largest growth rate of RT instability in the linearized stratified MRT problem. Thus we have the following conclusion.

#### Proposition 3.1

*Under the assumptions of Theorem* 3.1, *there exists a pair of functions* $(\tilde{u},\tilde{q})\in(H^{1}_{\sigma}\cap H^{\infty})\times H^{\infty}$ *satisfying the boundary problem* (3.16) *with a largest growth rate* $\Lambda>0$ *constructed by* (3.36). *Moreover*, $\|\tilde{u}_{3}\|_{0}\|(\tilde{u}_{1},\tilde {u}_{2})\|_{0}|\tilde{u}_{3}|_{0}>0$.

To emphasize the dependence of $\beta(s)$, Λ, and $\mathcal {G}$ upon $\mathcal{M}$, we will denote them by $\beta(s,\mathcal {M})$, $\Lambda_{\mathcal{M}}$, and $\mathcal{G}_{\mathcal{M}}$, respectively. Obviously, we have $\Lambda_{\mathcal{M}}=\Lambda_{-\mathcal{M}}$. To complete the proof of Theorem 3.1, we further derive (3.24)–(3.25). To this end, we need the following auxiliary conclusions.

#### Lemma 3.3

*Let* *g*, *μ*, *and* *ρ* *be given*. *We further assume that* 〚ρ〛>0. *The following assertions hold*:

1. *Strict monotonicity*: *if* $0\leq\mathcal{M}_{1}<\mathcal{M}_{2}$, *then*
  3.37 $$ \beta(s,\mathcal{M}_{2})< \beta(s,\mathcal{M}_{1}). $$
  *for any given* $s>0$. *Moreover*,
  3.38 $$ \mathcal{G}_{\mathcal{M}_{1}}>\mathcal{G}_{\mathcal{M}_{2}}, $$
  *where*
  3.39 $$ \mathcal{G}_{\mathcal{M}_{i}}:=\sup\bigl\{ s\in\mathbb{R}|\beta(\tau ,\mathcal{M}_{i})>0\textit{ for any } \tau\in(0,s)\bigr\} \quad \textit{and}\quad \beta(\mathcal{G}_{\mathcal{M}_{i}},\mathcal{M}_{i})=0. $$
2. *Continuity*: *for given* $s>0$, $\beta(s,\mathcal{M})\in C^{0,1}_{\mathrm{loc}}[0,\mathcal{M}_{{c}})$ *with respect to the variable *$\mathcal{M}$.
  (3) *Estimate for* $\mathcal{G}_{\mathcal{M}}$: $\mathcal{G}_{\mathcal {M}}\leq{\lambda(\mathcal{M}_{c}^{2}-\mathcal{M}^{2})}/{\min\{\mu _{-},\mu_{+}\}}$, *where* $\mathcal{M}$ *is a constant*.

#### Proof

(1) Let $s>0$ fixed, and let $0\leq\mathcal{M}_{1}<\mathcal{M}_{2}$. Then by Lemma 3.1 there exist functions $\tilde {u}^{\mathcal{M}^{i}}\in H^{\infty}\cap\mathcal{A}$, $i=1$, 2, such that $|\tilde{u}^{\mathcal{M}^{i}}|_{0}\neq0$ and

$$ \beta(s,\kappa_{i}) =\Xi\bigl(\tilde{u} ^{\mathcal{M}^{i}}\bigr) -\lambda \bigl\Vert \mathcal{M}^{i}\partial_{3} \tilde {u}^{\mathcal{M}^{i}} \bigr\Vert _{0}^{2}, $$

where $\Xi ({\tilde{u}}^{M^{i}}):=g〚\rho 〛{\left|{\tilde{u}}_{3}^{M^{i}}\right|}_{0}^{2}-s{∥\sqrt{\mu }S({\tilde{u}}^{M^{i}})∥}_{0}^{2}/2$, and $\tilde {u}^{\mathcal{M}^{i}}_{3}$ denotes the third component of $\tilde {u}^{\mathcal{M}^{i}}$. By (3.19) and (3.20) we have

$$ 0< \bigl\vert \tilde{u}^{\mathcal{M}^{i}} \bigr\vert _{0}^{2} \lesssim { \bigl\Vert \mathcal {M}^{i}\partial_{3} \tilde{u}^{\mathcal{M}^{i}} \bigr\Vert ^{2}_{0}},\quad {i}=1,2, $$

and thus

$$ \begin{aligned} \beta(s,\mathcal{M}_{2})\leq \beta(s, \mathcal{M}_{1})+\lambda\bigl(\mathcal{M}_{1}^{2}- \mathcal {M}_{2}^{2}\bigr) \int \bigl\vert \partial_{3}\tilde{u}^{\mathcal{M}_{2}} \bigr\vert ^{2}\,{d}y < \beta(s,\mathcal{M}_{1}). \end{aligned} $$

This yields the desired conclusion (3.37). Following the argument of (2.18), we easily get (3.38).

(2) Let $s>0$ be fixed. We choose bounded intervals $[b_{3},b_{4}]\subset (0,\infty)$ and $[b_{5},b_{6}]\subset[0,\mathcal{M}_{{c}})$ such that $s\in[b_{3},b_{4}]$. Then, for a given constant $s>0$ and any $\mathcal {M}\in[b_{5},b_{6}]$, there is a function $\tilde{w}^{\kappa}\in\mathcal {A}$ satisfying $\beta(s,M)=\Xi(\tilde{w} ^{\mathcal{M}}) -\lambda\|\mathcal{M}\partial_{3} \tilde {w}^{\mathcal{M}}\|_{0}^{2}$. Thus, in view of the monotonicity of $\beta (s,\mathcal{M})$, we know that

$$ \begin{aligned} \beta(b_{4},b_{6})+b_{3} \Phi\bigl(\tilde{w}^{\mathcal{M}}\bigr)/2&\leq\beta (b_{4}, \mathcal{M})+b_{3}\Phi\bigl(\tilde{w}^{\mathcal{M}}\bigr)/2\leq {E}_{0}\bigl(\tilde{w}^{\mathcal{M}}\bigr)-(s/2)\Phi\bigl( \tilde{w}^{\mathcal{M}}\bigr) \\ &\leq \beta(s/2,\mathcal{M})\leq\beta(b_{3}/2,\mathcal{M})\leq \beta(b_{3}/2,b_{5}), \end{aligned} $$

which, together with Korn’s inequality, yields

$$\begin{aligned} \bigl\Vert \partial_{3} \tilde{w}^{\mathcal{M}} \bigr\Vert _{0}^{2} &\leq \frac{c}{2} \bigl\Vert S\bigl(\tilde{w}^{\mathcal{M}}\bigr) \bigr\Vert _{0}^{2} \leq\frac{c}{\min\{\mu_{-},\mu_{+}\} }\Phi\bigl( \tilde{w}^{\mathcal{M}}\bigr) \\ &\leq2 \bigl(\beta(b_{3}/2,b_{5})-\beta(b_{4},b_{6}) \bigr)\frac{c}{b_{3}\min\{\mu_{-},\mu_{+}\}}:=K' \quad \mbox{for any } \mathcal{M} \in[b_{5},b_{6}]. \end{aligned} $$

Thus, for any $\mathcal{M}_{1}$, $\mathcal{M}_{2}\in[b_{5},b_{6}]$,

$$\begin{aligned} \beta(s,\mathcal{M}_{1})-\beta(s, \mathcal{M}_{2})&\leq \Xi\bigl(\tilde {w}^{\mathcal{M}_{1}}\bigr)-\lambda \bigl\Vert \mathcal{M}_{1}\partial_{3}\tilde {w}^{\mathcal{M}_{1}} \bigr\Vert _{0}^{2}- \bigl(\Xi\bigl( \tilde{w}^{\mathcal{M}_{1}}\bigr)-\lambda \bigl\Vert \mathcal {M}_{2} \partial_{3}\tilde{w}^{\mathcal{M}_{1}} \bigr\Vert _{0}^{2} \bigr) \\ &\leq \lambda K' \vert \mathcal{M}_{2}+ \mathcal{M}_{1} \vert \vert \mathcal {M}_{2}- \mathcal{M}_{1} \vert . \end{aligned} $$

Reversing the role of the indices 1 and 2 in the derivation of the above inequality, we obtain the same boundedness with the indices switched. Therefore, we deduce that

$$ \begin{aligned} \bigl\vert \beta(s,\mathcal{M}_{1})- \beta(s,\mathcal{M}_{2}) \bigr\vert \leq \lambda K' \vert \mathcal{M}_{2}+\mathcal{M}_{1} \vert \vert \mathcal{M}_{1}-\mathcal{M}_{2} \vert , \end{aligned} $$

which yields $\beta(s,\mathcal{M})\in C^{0,1}_{\mathrm{loc}}[0,\mathcal{M}_{{c}})$.

(3) Recalling the definition of $\mathcal{M}_{ c}$, we see that

$$\begin{array}{r} g〚\rho 〛|{\tilde{w}}_{3}{|}_{0}^{2}\leq \lambda {∥M_{c}\partial_{3}\tilde{w}∥}_{0}^{2}\;\text{for any }\tilde{w}\in A. \end{array}$$

By Lemma 3.1, for any given $\mathcal{M}$, there exists a nonzero function $\tilde{w}^{\mathcal{M}}\in\mathcal {A}\cap H^{\infty}$ such that

$$\beta (s,M)=g〚\rho 〛|{\tilde{w}}_{3}^{M}{|}_{0}^{2}-\lambda {∥M\partial_{3}{\tilde{w}}^{M}∥}_{0}^{2}-\frac{s}{2}{∥\sqrt{\mu }S\left({\tilde{w}}^{M}\right)∥}_{0}^{2}.$$

Moreover,

$$\bigl\Vert S\bigl(\tilde{w}^{\mathcal{M}}\bigr) \bigr\Vert _{0}^{2}=2 \bigl\Vert \tilde{w}^{\mathcal{M}} \bigr\Vert _{0}^{2}. $$

Thus

$$ \beta(s,\mathcal{M}) \leq\bigl(\lambda\bigl(\mathcal{M}_{{c}}^{2}- \mathcal {M}^{2}\bigr)-s\min\{\mu_{-},\mu_{+}\}\bigr) \bigl\Vert \partial_{3}\tilde{w}^{\mathcal{M}} \bigr\Vert _{0}^{2}. $$

Thus, if $\beta(s,\mathcal{M})\geq0$, then

$$ \begin{aligned} s\leq\lambda\bigl(\mathcal{M}_{{c}}^{2}- \mathcal{M}^{2}\bigr)/\min\{\mu_{-},\mu _{+}\}. \end{aligned} $$

This means that $\mathcal{G}_{\mathcal{M}}\leq\lambda(\mathcal {M}_{{c}}^{2}-\mathcal{M}^{2})/\min\{\mu_{-},\mu_{+}\}$ by the definition of $\mathcal{G}_{\mathcal{M}}$ in (3.39). This completes the proof. □

Now we are in position to show the properties (3.24) and (3.25) of $\Lambda_{\mathcal{M}}$ by three steps.

First, by the first assertion in Lemma 3.3 we can follow the argument of (2.20) to get the conclusion that, for given two constants $\mathcal{M}_{1}$ and $\mathcal{M}_{2}$ satisfying $0\leq\mathcal{M}_{1}<\mathcal{M}_{2}<\mathcal {M}_{{c}}$, it holds that

3.40 $$ \begin{aligned} \Lambda_{\mathcal{M}_{1}}> \Lambda_{\mathcal{M}_{2}}\quad \mbox{for }0\leq\mathcal{M}_{1}< \mathcal{M}_{2}. \end{aligned} $$

Second, by the monotonicity of $\Lambda_{\mathcal{M}}$ with regard to $\mathcal{M}$ and the monotonicity of $\alpha(s,\mathcal{M})$ we can follow the argument of (2.21) to get that $\Lambda_{\mathcal{M}} \in C^{0}[0,\mathcal{M}_{{c}})$. Since $\Lambda_{\mathcal{M}}=\Lambda_{-\mathcal{M}}$, we also have $\Lambda_{\mathcal{M}} \in C^{0}(-\mathcal{M}_{{c}},0]$. Thus we have

3.41 $$ \Lambda_{\mathcal{M}} \in C^{0}(-\mathcal{M}_{{c}}, \mathcal{M}_{{c}}). $$

Third, noting that $\Lambda_{\mathcal{M}}\in(0,\mathcal {G}_{\mathcal{M}})$, we immediately get

3.42 $$ \begin{aligned} \Lambda_{\mathcal{M}}\leq \lambda \bigl(\mathcal{M}_{{c}}^{2}-\mathcal {M}^{2}\bigr)/ \min\{\mu_{-},\mu_{+}\} \end{aligned} $$

by the third conclusion in Lemma 3.3. Consequently, we complete the proof of Theorem 3.1 from (3.40)–(3.42) and Proposition 3.1.

## Conclusion

In this paper, we prove that, if $\kappa<\kappa_{c}$, then there exists an unstable solution to the linearized stratified VRT problem with a largest growth rate. Moreover, the largest growth rate decreases from a positive constant to 0 as *κ* increases from 0 to $\kappa_{c}$. Moreover, we further extend the obtained results in the linearized stratified VRT problem to the linearized stratified MRT problem.
